# Sustainability of the rice-crayfish co-culture aquaculture model: microbiome profiles based on multi-kingdom analyses

**DOI:** 10.1186/s40793-022-00422-4

**Published:** 2022-05-22

**Authors:** Xue Zhu, Lei Ji, Mingyue Cheng, Huimin Wei, Zhi Wang, Kang Ning

**Affiliations:** 1 Key Laboratory of Molecular Biophysics of the Ministry of Education, Hubei Key Laboratory of Bioinformatics and Molecular-Imaging, Center of AI Biology, Department of Bioinformatics and Systems Biology, College of Life Science and Technology, Huazhong University of Science and Technology, Wuhan, 430074 Hubei China; 2 Key Laboratory for Environment and Disaster Monitoring and Evaluation of Hubei, Innovation Academy for Precision Measurement Science and Technology, Chinese Academy of Sciences, Wuhan, 430077 China; 3 University of Chinese Academy of Sciences, Beijing, 100049 China

**Keywords:** Multi-kingdom, Rice-crayfish co-culture model, Aquaculture models, Co-occurrence network, Horizontal gene transfer

## Abstract

While the rice-crayfish culture (RCFP) model, an important aquaculture model in Asia, is generally considered a sustainable model, its sustainability in terms of microbial community profiles has not been evaluated. In this study, multi-kingdom analyses of microbiome profiles (i.e., bacteria, archaea, viruses, and eukaryotes) were performed using environmental (i.e., water and sediment) and animal gut (i.e., crayfish and crab gut) microbial samples from the RCFP and other aquaculture models, including the crab-crayfish co-culture, crayfish culture, and crab culture models, to evaluate the sustainability of the RCFP systematically. Results showed that RCFP samples are enriched with a distinct set of microbes, including *Shewanella*, *Ferroplasma*, *Leishmania*, and *Siphoviridae*, when compared with other aquaculture models. Additionally, most microbes in the RCFP samples, especially microbes from different kingdoms, were densely and positively connected, which indicates their robustness against environmental stress. Whereas microbes in different aquaculture models demonstrated moderate levels of horizontal gene transfer (HGT) across kingdoms, the RCFP showed relatively lower frequencies of HGT events, especially those involving antibiotic resistance genes. Finally, environmental factors, including pH, oxidation–reduction potential, temperature, and total nitrogen, contributed profoundly to shaping the microbial communities in these aquaculture models. Interestingly, compared with other models, the microbial communities of the RCFP model were less influenced by these environmental factors, which suggests that microbes in the latter have stronger ability to resist environmental stress. The findings collectively reflect the unique multi-kingdom microbial patterns of the RCFP model and suggest that this model is a sustainable model from the perspective of microbiome profiles.

**Supplementary Information:**

The online version contains supplementary material available at 10.1186/s40793-022-00422-4.

## Background

Aquaculture products are among the most important sources of high‐quality, low-calorie protein [1–3]. In China, freshwater aquaculture products are the primary export of aquatic animal products, contributing ~ 60% yields of the total aquaculture product [4]. Sustainable models have been developed to produce more food from aquaculture using limited resources and with lower environmental impacts; indeed, co-aquaculture models are considered highly suitable for this purpose [5–7]. However, the definition of a sustainable model is mostly based on experience and lacks systematic evaluation protocols. Because microbial communities play a basic material and energy cycle driving role in various ecosystems [8–10], evaluating the sustainability of different aquaculture models in terms of their microbial profiles is necessary. The sustainability of an aquaculture model at the microbial community level could be represented and measured by the active interactions of microbes in the community, the frequency of gene transfer, especially of antibiotic resistance genes (ARGs), and ability of the communities to withstand environmental stress.

Crayfish farming is an important form of aquaculture in Asia, because crayfish is an excellent source of protein and essential amino acids [11]. Efforts to improve the sustainability of crayfish farming have led to the development of the rice-crayfish co-culture (RCFP) model. This model is a dominant model of crayfish farming and contributes ~ 90% of the total crayfish production [12] by taking advantage of the synergistic effects of co-cultured species [13]. It also improves rice yields and produces extra-economic profits [14]. In the RCFP model, alternating water and lower inputs of pesticides and chemical residues provide a green production environment (e.g., higher water quality, soil fertility, and dissolved oxygen contents), thereby reducing the risk of disease [13–16]. However, how the microbial profile of this model differs from that of other aquaculture models, as well as its robustness against the surrounding environment, is as yet unknown.

The microbial communities in an aquaculture co-culture model usually consist of bacteria, archaea, viruses, and eukaryotes. Further, their interactions include predation (e.g., some protists feed on bacteria), pathogenicity (e.g., microbes could interact with pathogens), and parasitism (e.g., some viruses live by parasitizing bacteria) [17]. These multi-kingdom species and their interactions jointly maintain the stability of the microbial community. However, the current knowledge on aquaculture microbial communities is mainly based on bacterial, fungal, or viral communities or the combination of two kingdoms [6, 18–20]. Studies profiling aquaculture microbial communities from the perspective of multiple kingdoms, including bacteria, archaea, viruses, and eukaryotes, are scarce. These knowledge gaps limit our in-depth understanding of microbial profiles in aquaculture models at the multi-kingdom level.

Aquaculture microbial communities are strongly influenced by environmental factors, including human activities, which often manifests as an increase in antimicrobial resistance [21]. When ARGs are transmitted into human-associated pathogens, they may pose a great environmental risk [22]. Many ARGs and other potentially harmful functional genes are spread through the environment by horizontal gene transfer (HGT) [23–25]. However, the current knowledge on HGT events in aquaculture models, especially those involving multi-kingdom microbes and ARGs, is limited. Moreover, the combined influence of multiple environmental factors on microbial communities in different aquaculture models remains unclear.

**Fig. 1:**
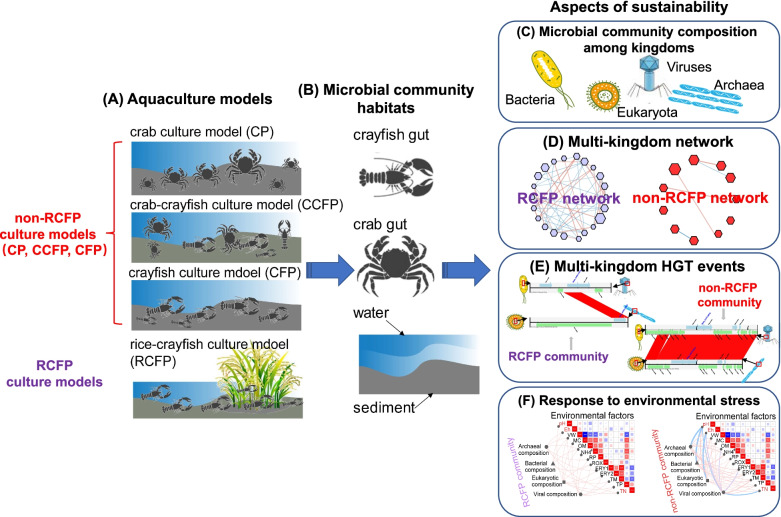
The workflow for systematically assessing the sustainability of the RCFP and other aquaculture models from the perspective of aquaculture model-specific microbial community patterns, multi-kingdom interactions, horizontal gene transfer (HGT) events, and environmental factors driving microbial community divergence. **A** Water, sediment, crayfish gut, and crab gut samples were collected from four representative aquaculture models: crab-crayfish culture model (CCFP), crab culture model (CP), crayfish culture model (CFP), and rice- crayfish co-culture model (RCFP). **B** The inhabiting habitats for the microbial community. **C** The aquaculture model-specific microbial community patterns of water, sediment, crayfish gut, and crab gut habitats. **D** Comparison of multi-kingdom interactions (bacteria, archaea, viruses, and eukaryotes) between RCFP and other aquaculture models in water, sediment, and crayfish gut habitats, respectively. **E** HGT events detecting across kingdoms between RCFP and other aquaculture models in water, sediment, and crayfish gut habitats, respectively. **F** The response of the multi-kingdom microbial community to environmental factors

In the present study, we analyzed the microbial communities of various aquaculture models to achieve a systematic assessment of the sustainability of the RCFP and other aquaculture models (Fig. 1). We focused on aquaculture model-specific microbial community patterns (Fig. 1C), multi-kingdom interactions (Fig. 1D), HGT events (Fig. 1E), especially those involving ARGs, and environmental factors driving microbial community divergence (Fig. 1F). To this end, we collected environmental (i.e., water and sediment) and animal gut (i.e., crayfish and crab gut) microbial samples from the crab-crayfish culture (CCFP), crab culture (CP), crayfish culture (CFP), and RCFP models (Fig. 1A, B). Specifically, we aim to answer four questions: (1) What are the distinct microbial community patterns of different aquaculture models at the multi-kingdom level? (2) How do bacteria, archaea, viruses, and eukaryotes interact with each other in different culture models? (3) How do HGT events, especially those involving ARGs, differ among different aquaculture models? (4) How do environmental factors influence the microbial communities in different aquaculture models? We found that the unique microbial profiles of the RCFP model maintain its stability when faced with environmental pressure, as reflected by its water, sediment, and crayfish gut microbial communities. The results confirm that the RCFP model is a sustainable model from the perspective of microbiome profiles and provide new insights into sustainable aquaculture.

## Methods

### Sample collection

**Fig. 2:**
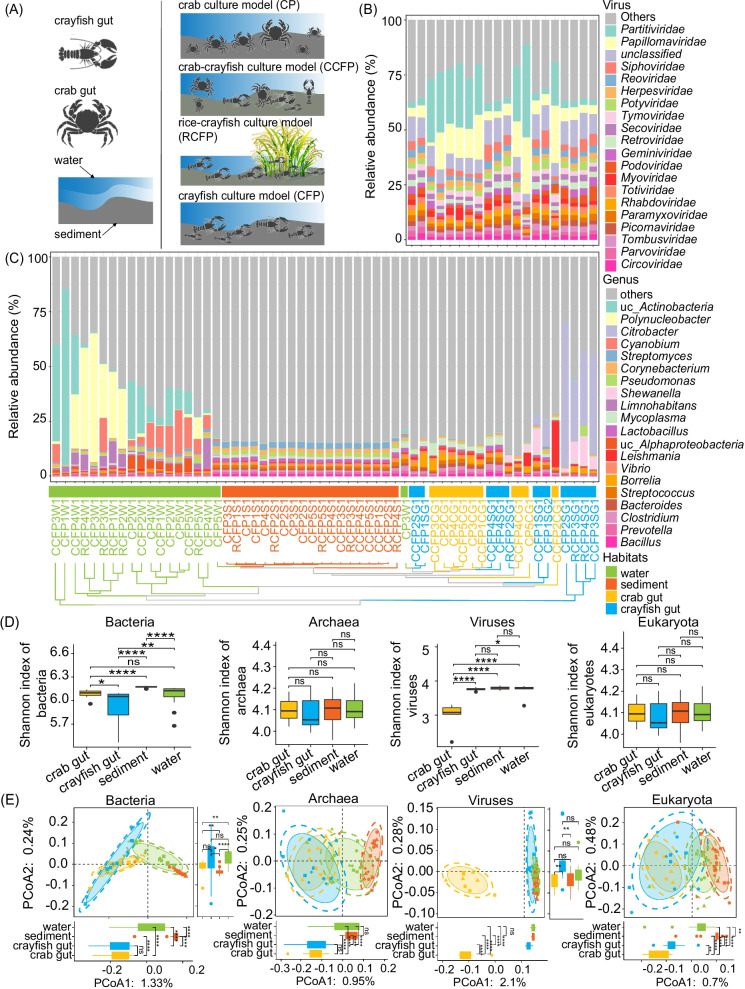
Microbial community composition and diversity. **A** Overview of the sampling habitats and aquaculture models. **B** Viral community composition. **C** Bacterial community composition at the genus level. Unweighted paired-group method with arithmetic means (UPGMA)-based hierarchical clustering (Bray–Curtis distance) was used to analyze the microbial community structure. Each column of Fig. 1B is vertically aligned to the columns of Fig. 1C. **D** Bacterial, archaeal, viral, and eukaryotic diversities across the crab gut, crayfish gut, sediment, and water habitats. **E** Comparison of microbial diversity across habitats via PCoA using Jaccard coefficients as the distance measurement. The 90% confidence intervals of each group are also shown in the background. Samples and tree branches colored in green, orange, blue, and yellow represent water, sediment, crayfish gut, and crab gut samples, respectively. CP: crab culture model; CFP: crayfish culture model; CCFP: crab-crayfish mixed culture model; RCFP rice-crayfish co-culture model; “uc’’: unclassified. “*”:*p* < 0.1; “**”:*p* < 0.05; “***”:*p* < 0.01; “****”:*p* < 0.001; ns: not significant

Samples from water, sediment, crayfish, and crab were collected from Honghu farms (29.92° N, 113.49° E), in Hubei Province, China. A total of 20 water and 20 sediment samples were collected from four different types of aquaculture models, including the CCFP, CFP, CP, and RCFP models (Fig. 1A, B). Five parallel samples were collected for each culture model (Figs. 1A, B, 2A and Additional file 1). The water and sediment samples were divided into two parts. One part was used to measure the effects of environmental factors according to our previous study [26]. The other part was utilized for metagenomic sequencing. Ten crab samples were collected from the CP (*n* = 5) and CCFP (*n* = 5) models, and 17 crayfish samples were collected from the CCFP (*n* = 5), CFP (*n* = 6), and RCFP (*n* = 6) models. The intestinal contents of the animals were aseptically extracted using the conventional anatomical method and placed in a sterile centrifuge tube (5 mL). In total, 67 samples were prepared and stored at -80 ℃ for metagenomic sequencing.

### Sample processing and sequencing

The total DNA of water filter membranes and sediment samples was extracted using the FastDNA™ SPIN Kit (MP, USA) following the manufacturer’s instructions. DNA extraction from crab gut and crayfish gut samples (~ 1 g) was performed using the QIAamp DNA Stool Mini Kit (Qiagen, Germany). The extracted DNA was treated using the NEBNext Ultra DNA Library Prep Kit for Illumina (NEB, USA) for whole-genome amplification and library preparation. Finally, paired-end sequencing was performed on an Illumina HiSeq X Ten platform. Except for 9 samples that could not be sequenced, 58 metagenomic samples were sequenced for metagenomic analysis (Additional file 1: Table S1). A total of 2.52 billion pair-end reads of raw sequencing data (Additional file 1: Table S2) were first evaluated using FastQC (version 0.11.6) [27]. Low-quality reads and adaptors were then trimmed by Trimmomatic (version 0.38) [28] to eliminate reads less than 100 bp in length, adapters, leading or trailing bases with Phred base quality (BQ) scores of < 20, and strings of every five bases with an average BQ score of < 25. After quality control, we obtained 2.41 billion high-quality pair-end reads (Additional file 1: Table S2).

### Microbial classification and diversity

To obtain the taxonomical composition of different models, we annotated high-quality reads (94.65% of the raw reads) from each sample by using MetaPhlAn2 (version 2.6.0) [29]. For each kingdom annotation, we filtered other kingdoms using “– ignore_bacteria – ignore_archaea – ignore_viruses – ignore_eukaryotes” items. Alpha diversity based on the Shannon index was determined using the diversity() function in R “vegan” package (version 2.6-2). Principle coordinate analysis (PCoA) based on Jaccard coefficients as a distance measurement was used to cluster samples according to their group with 90% confidence intervals.

### Detection of indicator microbes for different culture models

The indicator value of microbes in each culture model was calculated to characterize aquaculture model-specific microbial compositions by using the indval() function in R “labdsv” package (version 2.0-1) [30]; here, the frequency and relative abundance of each microbe were considered. In our study, only the microbes of an aquaculture model with the highest indicator values at *p* < 0.05 were considered indicator microbes for this model.

### Measurement and analysis of environmental factors

Environmental factors influencing the water and sediment samples were determined following our previously published study [26]. These environmental factors included physicochemical factors, such as temperature, total nitrogen (TN), total phosphorus (TP), sediment volume-weight (Sed_VM), and moisture content (MC), as well as antibiotic factors, such as roxithromycin (ROX), erythromycin (ERY), and erythromycin derivative 1 (ERY1). In total, we detected 18 physicochemical data and 5 antibiotic factors for water samples, and 9 physicochemical factors and 4 antibiotic factors for sediment samples (Additional file 1: Figs. S1, S2).

### Analysis of multi-kingdom co-occurrence networks

Microbes with a relative abundance of ≥ 2.00% and coverage of > 20% samples were selected for multi-kingdom interaction analysis to investigate microbial correlations among bacteria, archaea, viruses, and eukaryotes in different culture models. We used Spearman’s correlation analysis to calculate correlations among the four kingdoms. Only Spearman correlations of ≥ 0.65 or ≤ − 0.65 with *p* < 0.05 were considered as strong correlations and visualized in Cytoscape (version 3.8.1) [31].

### Analysis of horizontal gene transfer events

We investigated variations in ARGs and HGT events across different habitats and aquaculture models. We mainly used two methods to detect genes transferred among different kingdoms in water, sediment, and crayfish gut. In the first method, ARGs were detected using DeepARG (version 1.0.2) [32] and then mapped to microbial contigs using BLASTN (version 2.7.1+) with “-task megablast -evalue 1e-10.” These contigs were annotated for taxonomy assignment using Kraken2 (version 2.0.8-beta) [33]. In the second method, the genes involving HGT events were detected using MetaCHIP (version 1.10.4) [34] with “-r pcofgs” to predict reference-independent HGT events at the community level. Here, high-quality bins were built using MetaWRAP (version 1.2.2) [35]. A total of 496 bins (0.90 Gb) with an average length of 1,806,454 per bin were assembled from the 58 contigs (4.80 Gb; an average length of a contig: 2306). Then, all bins obtained from the metagenomic data, their taxonomic classifications, and their group information were inputted into MetaCHIP.

### Analysis of the effect of environmental factors on microbial profiles

We used detrended correspondence analysis (DCA) to judge the major axis length. Because the lengths of the first four major axes were less than three, redundancy analysis (RDA) was used to reveal the effects of the physicochemical and antibiotic factors of water and sediment habitats on microbial community ordination using the R package “vegan” (version 2.6-2). All environmental factors were tested in RDA analysis using the envfit() function with 999 permutations in R “vegan” package (version 2.6-2), while those with *p* < 0.05 were considered as significant factors influencing the corresponding microbial community.

### Statistical analysis

Differences among the water, sediment, crayfish gut, and crab gut habitats, as well as the RCFP and other culture models (i.e., the non-RCFP group), were determined using the Wilcoxon test in R “stats” package (version 4.0.5). Variations in habitat among different aquaculture models were tested by analysis of variance with the least-significant difference test. The *p* value was adjusted using the Benjamini and Hochberg method.

## Results

### Differences in environmental and animal gut microbial community patterns at the multi-kingdom level

Microbial community samples from different habitats, including water, sediment, crayfish gut, and crab gut, were collected from the CCFP, CFP, CP, and RCFP models (Fig. 1A, B, and 2A). After demultiplexing, adaptor trimming, and quality control, 2.41 billion high-quality pair-end reads were used for downstream analysis (Additional file 1: Table S1 and Fig. S3).

The microbial community composition varied across the different habitats. Through multi-kingdom analysis, we detected an average abundance of 61.84% bacteria, 4.62% archaea, 30.13% viruses, and 2.94% eukaryotes in each sample. The proportion of clean reads that could be assigned to different taxonomic levels in each habitat was also provided in Additional file 1: Table S3. We also found that environmental (sediment and water) microbial communities were dominated by bacteria, whereas animal gut microbial communities of crayfish and crab were dominated by both bacteria and viruses (Fig. 2 and Additional file 1: Figs. S4–S6). Water and sediment habitats harbored more archaea than those of crab gut and crayfish gut habitats, whereas animal gut habitats contained more eukaryotes than those of water and sediment habitats (Additional file 1: Figs. S4–S6). All detected microbial taxa in each habitat across these four kingdoms were also provided in Additional file 1: Fig. S7 and Table S4. Unweighted paired-group method with arithmetic means (UPGMA) analysis illustrated that the water, sediment, crayfish gut, and crab gut samples were respectively clustered together (Fig. 2 and Additional file 1: Fig. S6), thereby indicating that the microbial community composition largely depends on the habitat. In terms of microbial alpha diversity, water and sediments showed higher bacterial and viral diversities compared with animal guts. However, the diversities of archaeal and eukaryotic organisms did not differ among these four habitats (Fig. 2D and Additional file 1: Fig. S8). Beta diversity also revealed that the animal gut microbial communities differed from those obtained from the environment (Fig. 2E).

### Indicator microbes across aquaculture models

We analyzed indicator microbes from different aquacultures. Representative microbes with indicator values above the threshold (*p* < 0.05) were considered indicator microbes of the CCFP, CFP, CP, and RCFP models (Additional file 1: Tables S5–S8). A total of 26 and 33 indicator microbes, including bacteria, archaea, viruses, and eukaryotes, were detected as indicators for the RFCP model in the water and sediment habitats, respectively (Additional file 1: Tables S5–S6); these microbes could explain most of the differences across different aquaculture models. Taking the sediment habitat as an example, the indicator microbes such as *Ferroplasma* (average abundance: 0.42%), *Streptococcus* (average abundance: 1.54%), and *Microviridae* (average abundance: 0.39%) were detected in the RCFP model, *Halopiger* (average abundance: 0.61%), *Pseudomonas* (average abundance: 1.97%), and *Halorubrum* (average abundance: 7.36%) were detected in the CCFP model, *Podospora* (average abundance: 1.06%), *Alcanivorax* (average abundance: 0.18%), *Verticillium* (average abundance: 1.09%), and *uc_Tymovirales* (average abundance: 0.18%) for the CFP model, and *Serinicoccus* (average abundance: 0.13%) and *Secoviridae* (average abundance: 3.10%) were detected in the CP model (Additional file 1: Table S6). These indicator microbes were most abundant in the respective aquaculture models and primarily belonged to the bacterial phyla Proteobacteria, Actinobacteria, Firmicutes, archaeal phylum Euryarchaeota, and/or eukaryotic phylum Ascomycota. We also detected 35, 3, and 3 indicator microbes (*p* < 0.05) in the RCFP, CFP, and CCFP models, respectively, for the crayfish gut habitat (Additional file 1: Table S7), 7 and 17 indicator microbes in the CP and CCFP models, respectively, for the crab gut habitat (Additional file 1: Table S8).

### Multi-kingdom co-occurrence networks in different aquaculture models

To compare multi-kingdom co-occurrence networks in the RCFP models with those in other aquaculture models, we categorized all samples into two groups, namely, RCFP and non-RCFP; here, the RCFP group includes samples from the RCFP, while the non-RCFP group includes samples from all other aquaculture models.

**Fig. 3:**
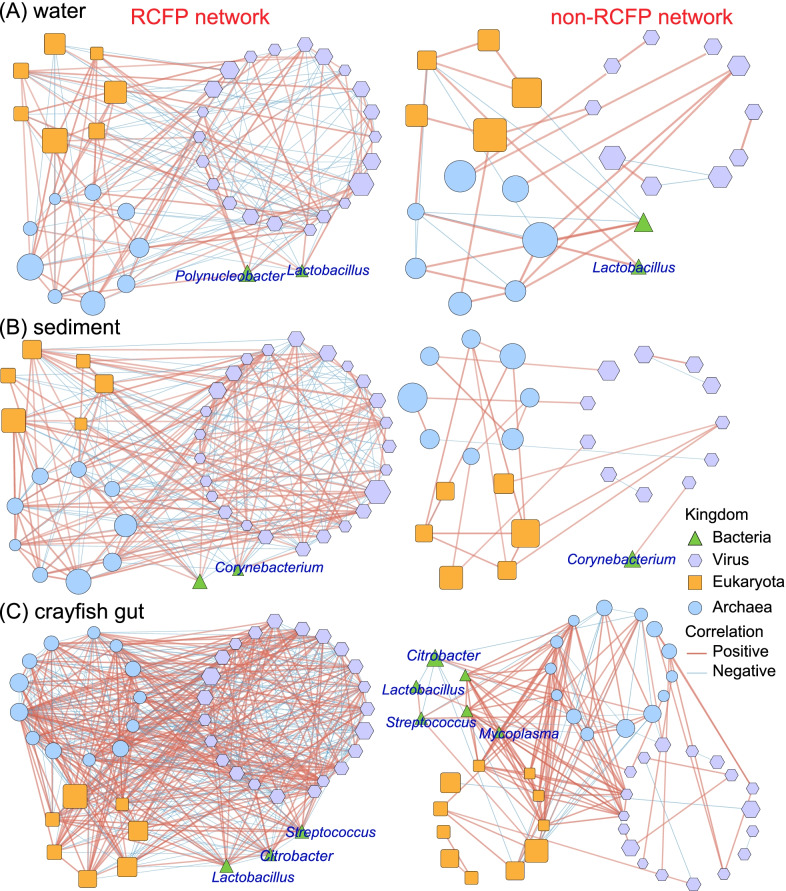
Multi-kingdom co-occurrence networks of samples in the RCFP and non-RCFP groups. Networks in **A** water, **B** sediment, and **C** crayfish gut habitats are presented, respectively. Microbes with a relative abundance of ≥ 2% and coverage of > 20% samples were used to construct the multi-kingdom co-occurrence networks. Only Spearman correlations of ≥ 0.65 or ≤ -0.65 with *p* < 0.05 were considered strong correlations and visualized in the network. The nodes in green, blue, purple, and orange represent bacteria, archaea, viruses, and eukaryotes, respectively. Red edges indicate positive correlations, whereas blue edges reflect negative correlations

The RCFP network was dominated by dense and positive multi-kingdom interactions in both water and sediment habitats. The RCFP model demonstrated higher network complexity in water habitats than the non-RCFP models. Specifically, in water habitat, the RCFP model showed 66.67% more nodes (40 vs. 24), 436.36% more edges (177 vs. 33), 83.3% greater network density (0.11 vs. 0.06), and 221.82% greater node connectivity (node degree: 8.85 vs. 2.75; all microbes were positively correlated) compared with the non-RCFP group. Details of the network properties obtained in water habitat were provided in Fig. 3A and Additional file 1: Table S9. We also observed that *Polynucleobacter* and *Lactobacillus* were positively correlated with most microbes, including bacteria, archaea, viruses, and eukaryotes, in the RCFP model, while the weak correlations were found in these microbes in the non-RCFP group in water habitat (Fig. 3A and Additional file 1: Fig. S9A). In sediment, although the number of microbes (46 nodes) used for RCFP network construction was ~ 6.52% fewer than that used for the non-RCFP group (49 nodes), the resulting network of the RCFP model was denser (0.134 vs. 0.04), and closer (average node degree: 11.00 vs. 2.00; the number of edges: 231 vs. 26) compared with that of the non-RCFP group (Fig. 3B, Additional file 1: Fig. S9B, and Additional file 1: Table S9). Moreover, compared with the RCFP group, we found weaker interactions between *Corynebacterium* and other microbes in the non-RCFP group. Taken, together, these results reflect the dense and close multi-kingdom interactions in the RCFP model in sediment habitats, consistent with the interactions found in water habitats.

The RCFP group also showed dense and positive networks across multiple kingdoms in the crayfish gut habitat. The network density of the RCFP group (network density: 0.24, node degree: 21.78) was approximately 2.5 times greater than that of the non-RCFP group (network density: 0.07, node degree: 6.25) (Fig. 3C, Additional file 1: Fig. S9C, and Additional file 1: Table S9). Compared with those in the RCFP network, the correlations among *Lactobacillus*, *Streptococcus*, and other microbes decreased in the non-RCFP group (Fig. 3C and Additional file 1: Fig. S9C). We found that *Citrobacter* was positively correlated with other kingdoms in the RCFP models, but these interactions were reduced or negative in the non-RCFP group. Besides, in the non-RCFP network, we determined that *Mycoplasma* was densely correlated with bacteria, archaea, viruses, and eukaryotes, which was not observed in the RCFP network (Fig. 3C and Additional file 1: Fig. S9C). Moreover, the network analysis has elucidated the specificities of the viral host in different aquaculture models (Additional file 1: Fig. S10). Collectively, these results indicated that the close and dense multi-kingdom interactions in the RCFP model may lead to the improved robustness of its microbial communities against environmental stress.

### Insights into HGTs within microbial communities

#### ARG transfer among multiple kingdoms

**Fig. 4:**
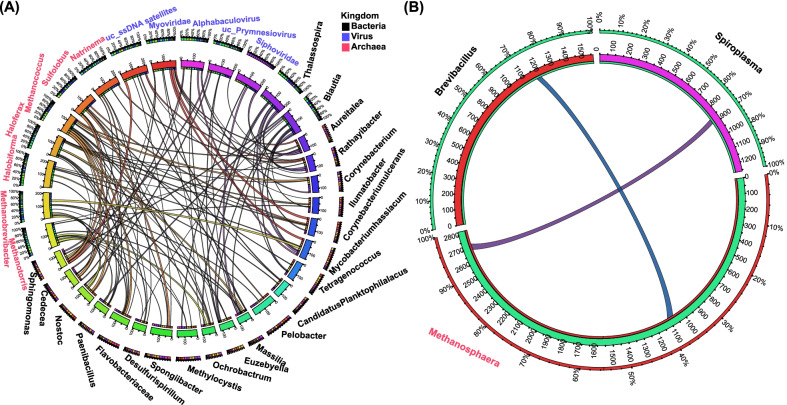
ARG-associated HGT events among kingdoms. ARGs transferring in **A** water and **B** crayfish gut habitats are shown. Each band in the inner or outer circle represents a microbe at the genus level, the name of which is colored according to the kingdom type: bacteria (black), archaea (red), and viruses (blue). Bands among bacterial, archaeal, and viral genomes mean the ARGs are involved in HGT events across kingdoms. Different band colors represent different microbes bearing genes involved in HGT events

The RCFP microbial communities demonstrated few ARG-associated HGT events in water and sediment habitats. We categorized samples into the RCFP and non-RCFP groups. HGT analysis showed that many ARGs are transferred among different microbes in water (5689 ARGs involved in HGT events, accounting for 14.00% of the total ARGs identified in water samples) and sediment (5091 ARGs involved in HGT events, accounting for 12.54% of the total ARGs identified in these samples) habitats (Fig. 4 and Additional file 1: Figs. S11–S12). In water habitats, 60 genes belonging to 8 ARG types were transferred across bacteria, archaea, and viruses, accounting for 34 microbes and 73 HGT events (Fig. 4A and Additional file 1: Fig. S11). In sediment habitats, four ARGs participated in 4 HGT events between bacteria and archaea (Additional file 1: Fig. S12). Among the aquaculture models studied, the RCFP model contributed the lower frequencies of HGT events both detected in water (1.4 HGT events per sample in the RCFP group; 4.7 HGT events per sample in the non-RCFP group) and sediment (zero HGT events per sample in the RCFP group; 0.29 HGT events per sample for the non-RCFP group) habitats (Fig. 4A and Additional file 1: Fig. S12).

RCFP microbial communities also contributed few ARG-involved HGT events in the crayfish gut habitat. Among 782 detected ARG-associated genes, three were annotated as MLS|macB and involved in two HGT events across kingdoms (Fig. 4B). Specifically, MLS|macB transferred between *Methanosphaera* and *Brevibacillus* in the RCFP group, as well as between *Methanosphaera* and *Spiroplasma* in both RCFP and non-RCFP groups. Together, these results implied that HGT events might play an important role in balancing the function of microbial communities and that RCFP microbial communities were less affected by ARG-associated HGT events than non-RCFP microbial communities.

### Functional genes participating in HGT events

The RCFP microbial community contributed few HGT events in both water and sediment habitats. Using MetaCHIP analysis, we detected 1.2 and 1.3 HGT events per sample in the RCFP and non-RCFP groups, respectively, in water habitats (Additional file 1: Fig. S13). In the RCFP group, *Polynucleobacter* contributed the largest number of HGT events, followed by *uc_Rhodocyclaceae* (Additional file 1: Fig. S13A). We also found that *Polynucleobacter* contributed to fewer HGT events (2 HGT events in all samples) in the non-RCFP group (Additional file 1: Fig. S13B). Moreover, we found that *Aeromonas*, a virulent and antibiotic-resistant pathogen [36, 37] that is harmful to aquaculture farming, participated in 3 HGT events in the non-RCFP group (Additional file 1: Fig. S13B).

In the crayfish gut habitat, the RCFP group contributed fewer HGT events compared with the non-RCFP group. Using MetaCHIP analysis, we detected only 4 HGT events across *Shewanella*, *Enterococcus*, and other unclassified microbes in all samples of non-RCFP model at the genus level (Additional file 1: Fig. S14). We also found that *Shewanella* and *Enterococcus* were among the top 10 bacterial hosts of ARGs (Additional file 1: Fig. S11D), and thus, could potentially accelerate ARG spreading. Taken together, the limited number of HGT events, especially ARG-associated HGT events, of RCFP microbial communities in both environmental and animal gut habitats suggested that the RCFP model was an environment-friendly aquaculture model.

### Effect of environmental factors on microbial communities

The RCFP was characterized by low nitrogen levels and antibiotic concentrations, especially in water habitats. In this study, the effects of environmental factors, including physicochemical parameters and antibiotics, on the microbial community structures were assessed. In water habitats, the RCFP model had higher salinity and oxidation–reduction potential (ORP) compared with other aquaculture models; by contrast, the concentrations of nitrate-nitrogen (NO_3_^−^-N), TN, turbidity, and erythromycin derivative 2 (EYR2) of the RCFP model were lower than those of other aquaculture models (*p* < 0.05) (Additional file 1: Fig. S1). These findings suggested low levels of nitrogen and antibiotic concentrations in the RCFP model. In sediment habitats, the pH of the RCFP model was higher compared with other aquaculture models (*p* < 0.05), but no significant differences in other environmental factors were found across the aquaculture models (Additional file 1: Fig. S2).

**Fig. 5:**
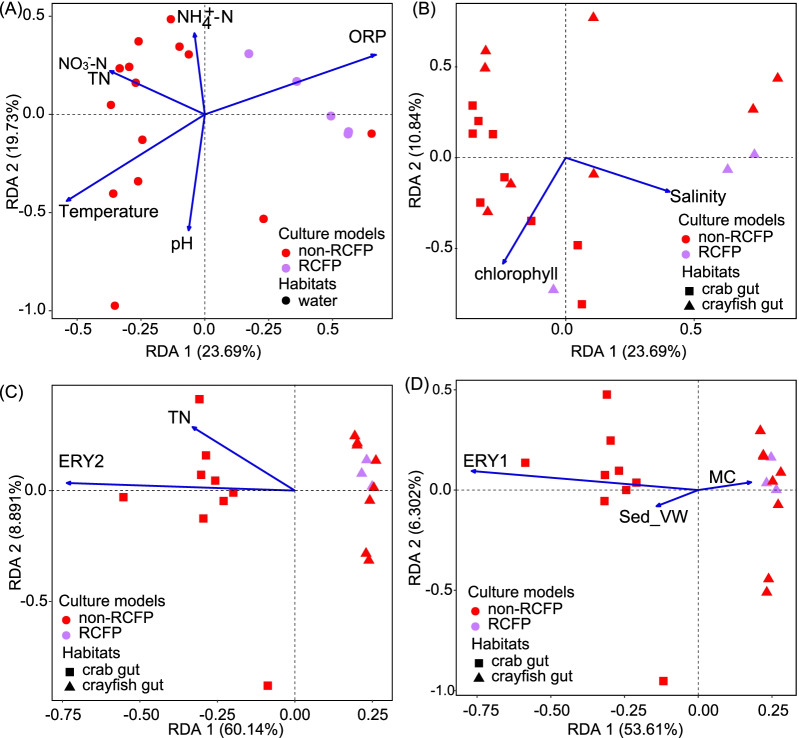
RDA analysis of the effect of environmental factors on microbial community structures. **A** Effect of water environmental factors on water bacterial communities. **B** Effect of water environmental factors on gut bacterial community compositions in crab and crayfish habitats, respectively. **C** Effect of water environmental factors on gut viral community compositions in crayfish and crab habitats, respectively. **D** Effect of sediment environmental factors on gut viral community compositions in crayfish and crab habitats, respectively. All environmental factors were tested in RDA analysis using the envfit() function with 999 permutations, and those with *p* < 0.1 were shown in the figure, while those with *p* < 0.05 were considered as significant factors influencing the corresponding microbial community. RCFP: rice-crayfish co-culture model; non-RCFP: all other aquaculture models excluding RCFP; ORP: oxidation–reduction potential; TN: total nitrogen; NH4^+^-N: ammonium nitrogen concentration; NO_3_^−^-N: nitrate-nitrogen concentration; Sed_VM: sediment volume-weight; MC: moisture content; ERY1: erythromycin derivative 1; ERY2: erythromycin derivative 2. Arrows indicate the contribution of environmental factors to the microbial community. Purple symbols indicate microbes in the RCFP group, and red symbols represent microbes in the non-RCFP group

The effects of environmental factors on microbial communities across different aquaculture models significantly differed. RDA revealed that, in water habitats, temperature (r^2^ = 0.510, *p* = 0.005) was a primary environmental factor affecting non-RCFP bacterial communities; other environmental factors, including pH and nitrogen level, also significantly influenced non-RCFP bacterial communities (Fig. 5A). By comparison, ORP (r^2^ = 0.546, *p* = 0.004) was the primary factor influencing the RCFP bacterial community (Fig. 5A). No other environmental factors significantly influenced (*p* < 0.05) the RCFP bacterial community. These environmental factors did not significantly affect the microbial community in sediment habitats across aquaculture models. Collectively, the results indicated that the microbial community of the RCFP was more stable under environmental stresses than those of other aquaculture models.

The microbial community of the RCFP model in the crayfish gut was only slightly affected by the studied environmental factors. In the crayfish gut habitat, chlorophyll (r^2^ = 0.268, *p* = 0.065) was the main factor influencing non-RCFP bacterial community structures (Fig. 5B). By contrast, we did not detect any environmental factor significantly affecting the RCFP microbial community (Fig. 5B, D). These results indicated that the microbial community in the RCFP model was more stable under environmental stresses in the crayfish gut than the microbial communities of other aquaculture models.

## Discussion

### Differences in microbial community patterns across aquaculture models

To the best of our knowledge, this study is the first to assess the microbial community of freshwater aquaculture wetlands from the perspective of multi-kingdoms. In terms of relative abundance, our research showed that bacteria are the most abundant microbes, followed by viruses, archaea, and eukaryotes. This result is consistent with previous studies on the microbial structure of the ocean environment at the multi-kingdom level [10, 38].

Specifically, our results showed that the RCFP model has a distinct set of microbes that play important ecological roles. In a water habitat, *Desulfotomaculum* is enriched in the RCFP model compared with other aquaculture models. *Desulfotomaculum arcticum* and *Desulfotomaculum geothermicum* are capable of degrading acetone and sulfate [39]. *Polydnaviridae*, a virus enriched in the non-RFCP model of water habitats, could encode virulence genes and infect the cells of the caterpillar host [40], which is harmful to crayfish farming. In sediment habitats, *Ferroplasma*, an indicator of RCFP, could remove inorganic sulfur compounds by mediating extracellular electron transfer [41]. *Verticillium* has been detected as an indicator of CFP and reported as a pathogen [42]. In the crayfish gut habitat, *Methanopyrus*, an obligate chemolithoautotrophic methanogen [43, 44], is enriched in the RCFP model and plays an important role in the remineralization and recycling of organic matter [45]. *Millerozyma* has been reported as a pathogen, and its enrichment in non-RCFP environments is detrimental to crayfish farming [46]. Such results suggest that compared with the RCFP model, other aquaculture models are enriched in many adverse microbes, which increases the opportunistic infection risk for crayfish [42, 46]. Moreover, in the RCFP model, many indicator microbes, including bacteria, archaea, viruses, and eukaryotes, play important roles in maintaining the balance of the aquaculture ecology [47, 48].

### Multi-kingdom interactions in the RCFP model

In this study, the multi-kingdom correlation network of the RCFP system was fairly complex, with a large number of nodes, edges, and degrees and high network density, which means it has a fairly stable microbial community structure, regardless of the habitat examined. Previous researchers showed that complex microbial network interactions reflect a stable microbial community structure [49, 50]. Because microbes are the basic driving force of environmental materials and energy flow [8–10], stable microbial structures often have better ability to resist external interferences, including human interference [51, 52]. Therefore, RCFP ecosystems can be considered to have good ecological sustainability. Such high ecological sustainability may be related to low exogenous pollution inputs (e.g., antibiotics and bait) and strong pollution self-purification ability (e.g., the purification role of rice) [12, 14, 52]. These results reveal the potential advantages of the RCFP model from the perspective of microbes.

### ARG exchange across kingdoms in the RCFP model

Given the extensive use and abuse of antibiotics in recent years, ARGs have gradually become a research hot spot [52–54]. In fact, ARGs are the inherent genes of microbes, and these genes have been found in permafrost microbes [53, 54]. In addition to resisting the stress of antibiotics, resistance genes often participate in information exchange among microbes and other important functions [55]. We found that ARG-associated HGT events play an important role in multi-kingdom microbial interactions in aquaculture models, i.e., multi-kingdom interactions existing in both environmental and animal habitats, and many of these interactions are mediated by ARGs. For example, in crayfish gut habitats, the ARG gene MLS|macB is transferred between *Methanosphaera* and *Brevibacillus* in the RCFP model. *Methanosphaera* is a methanogen [56], while *Brevibacillus* could degrade vinyl acetate in methanogenic reactors [57], which is beneficial for organic matter recycling [45]. Another example is *Polynucleobacter* in the RCFP model, which has been reported as the host of most ARGs [52, 58]. The functional genes of *Polynucleobacter* exert a positive selection process across different environments [59], which may help other microbes adapt to changing environmental conditions through HGT to acquire new functions [55]. Additionally, Rhodocyclaceae, which was also identified in the RCFP model, can degrade toluene and aromatic compounds under hypoxic [60]. The genes of these microbes participating in HGT events could help microbes rapidly adapt to changing environments through the acquisition of new functions [25, 61]. However, ARG-associated HGT events varied across different aquaculture models. For instance, *Citrobacter* was positively correlated with other kingdoms in the RCFP model, while interactions associated with *Citrobacter* were much less or negative in non-RCFP models. *Citrobacter* is an opportunistic pathogen in aquatic animals; in non-RCFP models, larger inputs of antibiotics and pesticides may induce the pathogenicity of *Citrobacter*, potentially causing high mortality in aquatic animals [62–64]. Collectively, these phenomena suggest that multi-kingdom interactions, especially those mediated by ARGs, shape the microbial composition across different aquaculture models.

Microbial antibiotic resistance and ARG pollution are considered among the most important global public threats in the twenty-first century [65]. In this study, we also discovered that non-RCFP aquaculture, particularly in the water habitat, was predicted to have a higher frequency of putative HGT events than that of the RCFP model. To seek high production and economics from aquaculture using fewer limited resources, a greater amount of pesticides were input into the non-RCFP model for treatment and prophylaxis [12, 66], which may induce the ARG spearing and contamination, thereby threatening the human and the surrounding environment [66, 67]. These antibiotics are directly entered into the water, while the water also acts as inhabiting filtering, thereby accumulating more ARGs [13, 14, 16, 68]. Our previous study also discovered more ARGs in the non-RCFP model in sediment compared with water habitats [52], while in this study, we found a higher frequency of HGT events in water habitat than that of sediment in the non-RCFP models, especially for the ARG-mediated HGT events, which might be attributed to a higher fluidity of water compared with sediment. Therefore, we believe that the lower frequency of ARGs present in the RCFP compared to non-RCFP, especially those transferred across microbes, is a strong indication of the sustainability of this model for several possible reasons. First, in the RCFP model, the presence of crayfish is linked to fewer antibiotic inputs [12, 14], and alternating water and high sediment quality could reduce the accumulation and spreading of ARGs [14]. Second, the water and sediment in the model could play as inhabiting filtering, such as ARG filtering, thereby reducing the occurrence of antibiotic resistance selection in the microbial community of crayfish [13, 14, 16, 68]. Finally, the high salinity in the RCFP model could reduce the relative abundance of ARGs [69]. Taken together, these phenomena suggest that the RCFP model is an environment-friendly and ecologically balanced aquaculture model.

### Effect of environmental factors on microbial communities

Microbial communities are affected by a variety of environmental factors [26, 70]. Previous studies mainly focused on the impact of different environmental factors on bacterial communities [71, 72], but an in-depth understanding of the impact of environmental factors on microbial communities at the multi-kingdom level in aquaculture models is still lacking. We found that, at the multi-kingdom level, the microbial communities of non-RCFP models are mainly affected by temperature, pH, and nitrogen level. This result is in line with the results of earlier multi-kingdom studies on the global ocean microbiome [10], which also showed that temperature and pH strongly influence microbial community structures and functions. In addition, previous studies reported that salinity, rather than temperature, could explain a significant portion of the distribution patterns of microbial communities [70]. In the present study, our results indicated that salinity (r^2^ = 0.28, *p* = 0.07) is indeed important but temperature (r^2^ = 0.510, *p* = 0.005) could explain a larger portion of the distribution patterns of the water microbial communities in non-RCFP aquaculture models. This finding may be attributed to higher temperatures generally being able to stimulate microbial growth and metabolism [73], which could increase the stability of the microbial community. Thus, temperature plays an important role in shaping the microbial composition structure in non-RCFP models. Our results indicated that RCFP microbial communities are less affected by environmental factors than non-RCFP microbial communities. In fact, we determined that the bacterial community of the RFCP model in water habitats was significantly influenced by ORP (r^2^ = 0.546, *p* = 0.004) only, mainly because ORP could adjust microbial metabolism via modulating the intracellular redox balance [74]. This result is consistent with a previous study that found that ORP exerts an important effect on the succession of both bacterial and fungal communities in the sludge composting process [74].

We further found that compared with the water microbial community, the crayfish gut microbial community is less affected by environmental factors, especially in the RCFP model. These results indicate that the microenvironment of animal guts is stable and fairly resistant to the effects of the external environment. Crayfish, for example, is able to resist exogenous environmental stress, which is related to the environmental adaptability of crayfish [16, 52]. In the RCFP model, the external environment appears to exert minimal effects on the microbial community in crayfish gut, because the rice-crayfish co-culture environment is relatively clean [12, 14, 52, 68] and can indirectly influence the crayfish gut microbial community [16].

## Conclusions

This study quantified the sustainability of the RCFP model at the multi-kingdom level on the basis of microbial communities. The sustainability of different aquaculture models has previously been examined but rarely quantified at this level. The multi-kingdom microbial profiles of different culture models were investigated from multiple aspects, including the complexity of the microbial community, network interactions, and the HGTs of functional genes, especially ARGs. Our results clearly illustrated that microbial communities from the RCFP model have unique indicator microbes, such as *Shewanella*, *Ferroplasma*, *Leishmania*, and *Siphoviridae*. Moreover, the RCFP microbes were densely and positively connected in the network, which suggests that these microbial communities are resilient to environmental stress. Our results further illustrated that HGT events of functional genes, especially ARGs, among bacteria, archaea, and viruses have lower frequencies in the RCFP model than that in other aquaculture models. Finally, environmental factors, such as pH, ORP, temperature, and TN, could substantially shape the microbial communities in the aquaculture environments, although the microbial communities from the RCFP model, especially in the crayfish gut, are less affected by these factors.

The results collectively indicate that the RCFP possesses specific patterns, including distinct microbial community structures, densely and positively connected microbial networks, lower frequency of HGT events, and robustness against environmental factors. The findings quantitatively confirm the sustainability of the RCFP culture model. Quantification of the sustainability of the RCFP on the basis of microbial profiles could provide a deeper understanding of the links between microbial communities in different aquaculture models and the environmental factors influencing these communities. This work represents one of the first studies on the microbial community of freshwater aquaculture wetlands from the multi-kingdom perspective and provides new insights into sustainable aquaculture.

## Supplementary Information

**Additional file 1:** Supplementary Materials.

## Data Availability

The raw metagenomic sequence data used in this study are available in the Genome Sequence Archive (GSA; https://ngdc.cncb.ac.cn/gsub/) database (GSA accession number: PRJCA009514).

## References

[1] Naylor RL, Hardy RW, Buschmann AH, Bush SR, Cao L, Klinger DH, Little DC, Lubchenco J, Shumway SE, Troell M (2021). A 20-year retrospective review of global aquaculture. Nature.

[2] Tidwell JH, Allan G: The role of aquaculture. In: Aquaculture production systems. 2012, p. 3–14.

[3] Fry JP, Mailloux NA, Love DC, Milli MC, Cao L (2018). Feed conversion efficiency in aquaculture: do we measure it correctly?. Environ Res Lett.

[4] Dong J (2020). The Fisheries Breau of Agriculture Ministry. China Fishery statistical yearbook.

[5] Bibbiani C, Campiotti A, Incrocci L, Pardossi A, Fronte B, Viola C (2017). Aquaponic as sustainable innovation for food production. Rivista di Studi Sulla Sostenibilita.

[6] Wei D, Xing C, Hou D, Zeng S, Zhou R, Yu L, Wang H, Deng Z, Weng S, He J, Huang Z (2021). Distinct bacterial communities in the environmental water, sediment and intestine between two crayfish-plant coculture ecosystems. Appl Microbiol Biotechnol.

[7] Napier JA, Haslam RP, Olsen R-E, Tocher DR, Betancor MB (2020). Agriculture can help aquaculture become greener. Nat Food.

[8] York A (2018). Marine biogeochemical cycles in a changing world. Nat Rev Microbiol.

[9] Hutchins DA, Fu F (2017). Microorganisms and ocean global change. Nat Microbiol.

[10] Sunagawa S, Coelho LP, Chaffron S, Kultima J, Labadie K, Salazar G, Djahanschiri B, Zeller G, Mende D, Alberti A (2015). Structure and function of the global ocean microbiome. Science.

[11] Venugopal V, Gopakumar K (2017). Shellfish: nutritive value, health benefits, and consumer safety. Comp Rev Food Sci Food Saf.

[12] Jiang Y, Cao C (2021). Crayfish–rice integrated system of production: an agriculture success story in China. A review. Agron Sustain Dev.

[13] Altieri MA (2004). Linking ecologists and traditional farmers in the search for sustainable agriculture. Front Ecol Environ.

[14] Jin T, Ge C, Gao H, Zhang H, Sun X (2020). Evaluation and screening of co-culture farming models in rice field based on food productivity. Sustainability.

[15] Leigh C, Stewart-Koster B, Sang NV, Truc LV, Hiep LH, Xoan VB, Tinh NTN, An T, Sammut J, Burford MA (2020). Rice-shrimp ecosystems in the Mekong Delta: linking water quality, shrimp and their natural food sources. Sci Total Environ.

[16] Liu Q, Long Y, Li B, Zhao L, Luo J, Xu L, Luo W, Du Z, Zhou J, Yang S (2020). Rice-shrimp culture: a better intestinal microbiota, immune enzymatic activities, and muscle relish of crayfish (*Procambarus clarkii*) in Sichuan Province. Appl Microbiol Biotechnol.

[17] Rohwer F, Vega Thurber R (2009). Viruses manipulate the marine environment. Nature.

[18] Sun F, Wang Y, Wang C, Zhang L, Tu K, Zheng Z (2019). Insights into the intestinal microbiota of several aquatic organisms and association with the surrounding environment. Aquaculture.

[19] Alfiansah YR, Hassenrück C, Kunzmann A, Taslihan A, Harder J, Gärdes A (2018). Bacterial abundance and community composition in pond water from shrimp aquaculture systems with different stocking densities. Front Microbiol.

[20] Zhang Z, Deng Q, Cao X, Zhou Y, Song C (2021). Patterns of sediment fungal community dependent on farming practices in aquaculture ponds. Front Microbiol.

[21] Schar D, Klein EY, Laxminarayan R, Gilbert M, Van Boeckel TP (2020). Global trends in antimicrobial use in aquaculture. Sci Rep.

[22] Zhao Z (2021). Comparison of microbial communities and the antibiotic resistome between prawn mono- and poly-culture systems. Ecotoxicol Environ Saf.

[23] Uluseker C, Kaster KM, Thorsen K, Basiry D, Shobana S, Jain M, Kumar G, Kommedal R, Pala-Ozkok I (2021). A review on occurrence and spread of antibiotic resistance in wastewaters and in wastewater treatment plants: mechanisms and perspectives. Front Microbiol.

[24] de Abreu VAC, Perdigão J, Almeida S (2020). Metagenomic approaches to analyze antimicrobial resistance: an overview. Front Genet.

[25] Groussin M, Poyet M, Sistiaga A, Kearney SM, Moniz K, Noel M, Hooker J, Gibbons SM, Segurel L, Froment A (2021). Elevated rates of horizontal gene transfer in the industrialized human microbiome. Cell.

[26] Han M, Dsouza M, Zhou C, Li H, Zhang J, Chen C, Yao Q, Zhong C, Zhou H, Gilbert JA (2019). Agricultural risk factors influence microbial ecology in Honghu Lake. Genom Proteom Bioinform.

[27] Brown J, Pirrung M, McCue LA (2017). FQC dashboard: integrates FastQC results into a web-based, interactive, and extensible FASTQ quality control tool. Bioinformatics.

[28] Bolger AM, Lohse M, Usadel B (2014). Trimmomatic: a flexible trimmer for Illumina sequence data. Bioinformatics.

[29] Truong DT, Franzosa EA, Tickle TL, Scholz M, Weingart G, Pasolli E, Tett A, Huttenhower C, Segata N (2015). MetaPhlAn2 for enhanced metagenomic taxonomic profiling. Nat Methods.

[30] De Cáceres M, Legendre P, Wiser SK, Brotons L (2012). Using species combinations in indicator value analyses. Methods Ecol Evol.

[31] Shannon P, Markiel A, Ozier O, Baliga NS, Wang JT, Ramage D, Amin N, Schwikowski B, Ideker T (2003). Cytoscape: a software environment for integrated models of biomolecular interaction networks. Genome Res.

[32] Arango-Argoty G, Garner E, Pruden A, Heath LS, Vikesland P, Zhang L (2018). DeepARG: a deep learning approach for predicting antibiotic resistance genes from metagenomic data. Microbiome.

[33] Wood DE, Lu J, Langmead B (2019). Improved metagenomic analysis with Kraken 2. Genome Biol.

[34] Song W, Wemheuer B, Zhang S, Steensen K, Thomas T (2019). MetaCHIP: community-level horizontal gene transfer identification through the combination of best-match and phylogenetic approaches. Microbiome.

[35] Uritskiy GV, DiRuggiero J, Taylor J (2018). MetaWRAP-a flexible pipeline for genome-resolved metagenomic data analysis. Microbiome.

[36] Zhong C, Han M, Yang P, Chen C, Yu H, Wang L, Ning K (2019). Comprehensive analysis reveals the evolution and pathogenicity of aeromonas, viewed from both single isolated species and microbial communities. mSystems.

[37] Igbinosa IH, Beshiru A, Odjadjare EE, Ateba CN, Igbinosa EO (2017). Pathogenic potentials of Aeromonas species isolated from aquaculture and abattoir environments. Microb Pathog.

[38] Lima-Mendez G, Faust K, Henry N, Decelle J, Colin S, Carcillo F, Chaffron S, Ignacio-Espinosa JC, Roux S, Vincent F (2015). Determinants of community structure in the global plankton interactome. Science.

[39] Frey J, Kaßner S, Schink B (2021). Two marine Desulfotomaculum spp. of different origin are capable of utilizing acetone and higher ketones. Curr Microbiol.

[40] Herniou EA, Huguet E, Thézé J, Bézier A, Periquet G, Drezen J-M (2013). When parasitic wasps hijacked viruses: genomic and functional evolution of polydnaviruses. Philos Trans R Soc Lond B Biol Sci.

[41] Ni G, Simone D, Palma D, Broman E, Wu X, Turner S, Dopson M (2018). A novel inorganic sulfur compound metabolizing ferroplasma-like population is suggested to mediate extracellular electron transfer. Front Microbiol.

[42] Seidl MF, Kramer HM, Cook DE, Fiorin GL, van den Berg GCM, Faino L, Thomma B (2020). Repetitive elements contribute to the diversity and evolution of centromeres in the fungal genus verticillium. MBio.

[43] Hook SE, Wright AD, McBride BW (2010). Methanogens: methane producers of the rumen and mitigation strategies. Archaea.

[44] Kurr M, Huber R, König H, Jannasch HW, Fricke H, Trincone A, Kristjansson JK, Stetter KO (1991). Methanopyrus kandleri, gen. and sp. Nov. represents a novel group of hyperthermophilic methanogens, growing at 110 °C. Arch Microbiol.

[45] Lyu Z, Shao N, Akinyemi T, Whitman WB (2018). Methanogenesis. Curr Biol.

[46] Hong SI, Suh YS, Kim HO, Bae IG, Shin JH, Cho OH (2018). Successful treatment of catheter related blood stream infection by millerozyma farinosa with micafungin: a case report. Infect Chemother.

[47] Li D, Ni H, Jiao S, Lu Y, Zhou J, Sun B, Liang Y (2021). Coexistence patterns of soil methanogens are closely tied to methane generation and community assembly in rice paddies. Microbiome.

[48] Huang X, Wang J, Dumack K, Liu W, Zhang Q, He Y, Di H, Bonkowski M, Xu J, Li Y (2021). Protists modulate fungal community assembly in paddy soils across climatic zones at the continental scale. Soil Biol Biochem.

[49] Yuan MM, Guo X, Wu L, Zhang Y, Xiao N, Ning D, Shi Z, Zhou X, Wu L, Yang Y (2021). Climate warming enhances microbial network complexity and stability. Nat Clim Change.

[50] Dougoud M, Vinckenbosch L, Rohr RP, Bersier LF, Mazza C (2018). The feasibility of equilibria in large ecosystems: a primary but neglected concept in the complexity-stability debate. PLoS Comput Biol.

[51] Price GW, Langille MGI, Yurgel SN (2021). Microbial co-occurrence network analysis of soils receiving short- and long-term applications of alkaline treated biosolids. Sci Total Environ.

[52] Ning K, Ji L, Zhang L, Zhu X, Wei H, Han M, Wang Z (2021). Is rice-crayfish co-culture a better aquaculture model: from the perspective of antibiotic resistome profiles. Environ Pollut.

[53] Qian X, Gunturu S, Guo J, Chai B, Cole JR, Gu J, Tiedje JM (2021). Metagenomic analysis reveals the shared and distinct features of the soil resistome across tundra, temperate prairie, and tropical ecosystems. Microbiome.

[54] Zhang S, Yang G, Hou S, Zhang T, Li Z, Liang F (2018). Distribution of ARGs and MGEs among glacial soil, permafrost, and sediment using metagenomic analysis. Environ Pollut.

[55] Brito IL (2021). Examining horizontal gene transfer in microbial communities. Nat Rev Microbiol.

[56] Fricke WF, Seedorf H, Henne A, Krüer M, Liesegang H, Hedderich R, Gottschalk G, Thauer RK (2006). The genome sequence of *Methanosphaera stadtmanae* reveals why this human intestinal archaeon is restricted to methanol and H2 for methane formation and ATP synthesis. J Bacteriol.

[57] Lara-Mayorga I, Durán-Hinojosa U, Arana-Cuenca A, Monroy-Hermosillo O, Ramírez-Vives F (2010). Vinyl acetate degradation by *Brevibacillus agri* isolated from a slightly aerated methanogenic reactor. Environ Technol.

[58] Bai Y, Ruan X, Xie X, Yan Z (2019). Antibiotic resistome profile based on metagenomics in raw surface drinking water source and the influence of environmental factor: a case study in Huaihe River Basin, China. Environ Pollut.

[59] Sangwan N, Zarraonaindia I, Hampton-Marcell JT, Ssegane H, Eshoo TW, Rijal G, Negri MC, Gilbert JA (2016). Differential functional constraints cause strain-level endemism in polynucleobacter populations. mSystems.

[60] Táncsics A, Farkas M, Szoboszlay S, Szabó I, Kukolya J, Vajna B, Kovács B, Benedek T, Kriszt B (2013). One-year monitoring of meta-cleavage dioxygenase gene expression and microbial community dynamics reveals the relevance of subfamily I.2.C extradiol dioxygenases in hypoxic, BTEX-contaminated groundwater. Syst Appl Microbiol.

[61] Wei Z, Feng K, Wang Z, Zhang Y, Yang M, Zhu YG, Virta MPJ, Deng Y (2021). High-Throughput single-cell technology reveals the contribution of horizontal gene transfer to typical antibiotic resistance gene dissemination in wastewater treatment plants. Environ Sci Technol.

[62] Jia K, Yang N, Zhang X, Cai R, Zhang Y, Tian J, Raza SHA, Kang Y, Qian A, Li Y (2020). Genomic, morphological and functional characterization of virulent bacteriophage IME-JL8 targeting *Citrobacter freundii*. Front Microbiol.

[63] Yan BL, Zhang XJ, Liang LG, Yang W, Yang JX (2012). Detection of cfa gene and isolation and identification of the pathogen *Citrobacter freundii* isolated from *Portunus trituberculatus*. J Fish China.

[64] Heo G-J, Hossain S, Wimalasena S (2016). Virulence factors and antimicrobial resistance pattern of *Citrobacter freundii* isolated from healthy pet turtles and their environment. Asian J Anim Vet Adv.

[65] Fair RJ, Tor Y (2014). Antibiotics and bacterial resistance in the 21st century. Perspect Medicin Chem.

[66] Pepi M, Focardi S (2021). Antibiotic-resistant bacteria in aquaculture and climate change: a challenge for health in the mediterranean area. Int J Environ Res Public Health.

[67] Nguyen AQ, Vu HP, Nguyen LN, Wang Q, Djordjevic SP, Donner E, Yin H, Nghiem LD (2021). Monitoring antibiotic resistance genes in wastewater treatment: current strategies and future challenges. Sci Total Environ.

[68] Braun G, Braun M, Kruse J, Amelung W, Renaud FG, Khoi CM, Duong MV, Sebesvari Z (2019). Pesticides and antibiotics in permanent rice, alternating rice-shrimp and permanent shrimp systems of the coastal Mekong Delta, Vietnam. Environ Int.

[69] Bhowmick UD, Bhattacharjee S (2018). Bacteriological, clinical and virulence aspects of aeromonas-associated diseases in humans. Pol J Microbiol.

[70] Hou D, Huang Z, Zeng S, Liu J, Wei D, Deng X, Weng S, He Z, He J (2017). Environmental factors shape water microbial community structure and function in shrimp cultural enclosure ecosystems. Front Microbiol.

[71] Dabadé DS, Wolkers-Rooijackers JCM, Azokpota P, Hounhouigan DJ, Zwietering MH, Nout MJR, den Besten HMW (2016). Bacterial concentration and diversity in fresh tropical shrimps (*Penaeus notialis*) and the surrounding brackish waters and sediment. Int J Food Microbiol.

[72] Logares R, Lindström ES, Langenheder S, Logue JB, Paterson H, Laybourn-Parry J, Rengefors K, Tranvik L, Bertilsson S (2013). Biogeography of bacterial communities exposed to progressive long-term environmental change. ISME J.

[73] Wang Z, Brown JH, Tang Z, Fang J (2009). Temperature dependence, spatial scale, and tree species diversity in eastern Asia and North America. Proc Natl Acad Sci U S A.

[74] Wang K, Mao H, Li X (2018). Functional characteristics and influence factors of microbial community in sewage sludge composting with inorganic bulking agent. Bioresour Technol.

