# Supplementary material for: Sustainability of the rice-crayfish co-culture aquaculture model: microbiome profiles based on multi-kingdom analyses
Source: Environ Microbiome. 2022 May 22;17:27. doi: 10.1186/s40793-022-00422-4 (PMC9124410; doi:10.1186/s40793-022-00422-4)
Supplement: Supplementary file 1 — Additional file 1: Supplementary Materials. [file 40793_2022_422_MOESM1_ESM.docx]

**Additional file 1: Supplementary Materials**

# Sustainability of the rice-crayfish co-culture aquaculture model: Microbiome profiles based on multi-kingdom analyses

Xue Zhu^1^, Lei Ji^1^, Mingyue Cheng^1^, Huimin Wei^2,3^, Zhi Wang^2,*^, Kang Ning^1,*^

^1^Key Laboratory of Molecular Biophysics of the Ministry of Education, Hubei Key Laboratory of Bioinformatics and Molecular-imaging, Center of AI Biology, Department of Bioinformatics and Systems Biology, College of Life Science and Technology, Huazhong University of Science and Technology, Wuhan 430074, Hubei, China

^2^Key Laboratory for Environment and Disaster Monitoring and Evaluation of Hubei, Innovation Academy for Precision Measurement Science and Technology, Chinese Academy of Sciences, Wuhan 430077, China

^3^University of Chinese Academy of Sciences, Beijing 100049, China

^*^Correspondence should be addressed to K.N (Email: ningkang@hust.edu.cn) and Z.W (Email: zwang@apm.ac.cn)

**Supplementary Note 1. *The experimental process of water and sediment samples.***

For water sampling, 1.5 L of water with a depth of 0.3-0.5  m was collected at each sampling site using a cylinder sampler. Approximately 1 L of water sample was used for physicochemical index and antibiotics measurement, and 500 mL of water sample was first prefiltered using a 20 *μ*m tulle to remove large particles and then re-filtered using a polycarbonate membrane with a pore size of 0.22 *μ*m (Tianjin Jinteng Experiment Equipment Co., Ltd, China). Then these membranes were placed in a sterile centrifuge tube (50 mL) and stored in -80 ℃ before metagenomic sequencing.

For sediment sampling, 20 surface sediment samples (0-5 cm) were collected using a grab sampler. Approximately 300 g sediment samples were collected and stored in drikold until transportation to the laboratory. Among these, 250 g sediment samples were used for physicochemical index and antibiotics measurement, and 50 g sediment samples were utilized for metagenomic sequencing.

**Supplementary Figure legends**

**Fig. S1. Comparison of water environmental factors among different aquaculture models.** The variation among different aquaculture models was tested by variance with the least-significant difference tests. The presence of different letters denoted significant differences (p < 0.05) among culture models, and the same letter indicated no significant difference. BGA-PC: blue-green alga phycocyanin chlorophyll; COD: chemical oxygen demand; DO: dissolved oxygen; fDOM: fluorescent dissolved organic matter; NH_4_^+^-N: ammonium nitrogen concentration; NO_2_^-^-N: nitrite-nitrogen concentration; NO_3_^−^-N: nitrate-nitrogen concentration; ORP: oxidation-reduction potential; orthoP: orthophosphate; SpCond: specific conductance; TN: total nitrogen; TP: total phosphorus; ERY: Erythromycin; ERY1: erythromycin derivative 1; ERY2: erythromycin derivative 2; ROX: roxithromycin; TM: Total macrolide.

**Fig. S2. Comparison of sediment environmental factors among different aquaculture models.** The variation among different types of culture models was tested by variance with the least-significant difference tests. The presence of different letters denoted significant differences (p < 0.05) among culture models and the same letter indicated no significant difference. NH_4_^+^-N: ammonium nitrogen concentration; RP: released phosphorus concentration; Eh: electric potential half cells; MC: moisture content; OM: proportion of organic matter; Sed_VM: volume-weight of sediment; TN: total nitrogen; TP: total phosphorus; ERY1: erythromycin derivative 1; ERY2: erythromycin derivative 2; ROX: roxithromycin; TM: Total macrolide.

**Fig. S3**. **Rarefaction carves of sequencing depth of 58 successfully sequenced samples collected from Honghu, Hubei province, China.** Each curve represents a sample. The x-axis represents the number of sequences, and the y-axis represents the number of detected species.

**Fig. S4. Microbial composition at kingdom level.** The horizontal bar colored in yellow, blue, orange, and green color represented the samples collected from crab gut, crayfish gut, sediment, and water habitat, respectively. Each vertical column represented a sample. Each color in a vertical column represents a kingdom.

**Fig. S5. Comparison of the relative abundance of kingdoms within and among habitats.** (A) Comparison of the relative abundance of Archaea, Bacteria, Eukaryota, Viruses, and Viroids to determine the dominated kingdom within a habitat. (B) Comparison of the relative abundance of a kingdom among carb gut, crayfish gut, sediment, and water habitat to detect the dominated kingdom among habitats. The variation among culture models was tested by variance with the least-significant difference tests. The presence of different letters denoted significant differences (p < 0.05) among culture models and the same letter indicated no significant difference.

**Fig. S6. Microbial composition among kingdoms.** (A) Archaea community. (B) Eukaryotic community. (C) Viral community. For archaea and eukaryotic composition were based on the top 20 genera. The horizontal bar colored in yellow, blue, orange and green color represented the samples collected from crab gut, crayfish gut, sediment, and water habitats, respectively. Each vertical column represented a sample. Others mean the microbes were not among the top 20 microbes. “uc’’: unclassified.

**Fig. S7. Core- and pan-taxa curves within each habitat.** The pan (the blue curve) and the core bacteria (the red curve) for crab gut samples (A), crayfish gut samples (B), sediment samples (C), and water samples (D), respectively. The pan (the blue curve) and the core viruses (the red curve) for crab gut samples (E), crayfish gut samples (F), sediment samples (G), and water samples (H), respectively. The pan (the blue curve) and the core archaea (the red curve) for crab gut samples (I), crayfish gut samples (J), sediment samples (K), and water samples (L), respectively. The pan (the blue curve) and the core eukaryotes (the red curve) for crab gut samples (M), crayfish gut samples (N), sediment samples (O), and water samples (P), respectively. The x-axis represents the number of samples, while y-axis means the number of detected core or pan taxa when adding a sample.

**Fig. S8. Comparison of microbial diversity among different types of culture models.** (A) Comparison of bacterial diversity among different culture models within each habitat. (B) Comparison of bacterial diversity between RCFP and non-RCFP within each habitat. (C) Comparison of viral diversity among crab gut, crayfish gut, water, and sediment samples based on Shannon index. (D) Comparison of viral diversity among different culture models within each habitat. (E) Comparison of archaea diversity among different culture models within each habitat. (F) Comparison of the archaea diversity between RCFP and non-RCFP within each habitat. (G) Comparison of eukaryotic diversity among different culture models within each habitat. (H) Comparison of eukaryotic diversity between RCFP and non-RCFP within each habitat. Water: colored in green; Sediment: colored in orange; crab gut: colored in yellow; crayfish gut: colored in blue. CCFP: crab-crayfish culture model; CFP: crayfish culture model; CP: crab culture model; RCFP: rice-crayfish culture model. The variations among culture models were tested through an analysis of variance with least-significant-difference tests. The presence of different letters denoted significant differences (p < 0.05) among culture models, and the same letter indicated no significant difference.

**Fig. S9. Multi-kingdom co-occurrence networks of samples in RCFP and non-RCFP groups with all microbe names annotated.** Networks in (A) water, (B) sediment, and (C) crayfish gut habitats are presented, respectively. Microbes with a relative abundance of ≥2% and coverage of >20% samples were used to construct the multi-kingdom co-occurrence networks. Only Spearman correlations of ≥0.65 or ≤−0.65 with p < 0.05 were considered strong correlations and visualized in the network. The nodes in green, blue, purple, and orange represent bacteria, archaea, viruses, and eukaryotes, respectively. Red edges indicate positive correlations, whereas blue edges reflect negative correlations.

**Fig. S10. The host distribution of viruses by querying the IMG/VR and RefSeq databases in crayfish gut habitat.** (A) The phylum-level bacterial host of virus in different aquaculture models by querying the IMG/VR and RefSeq database. (B) The potential phylum-level bacterial host of viruses. (C) The potential genus-level host of the virus. The genus-level hosts of viruses were from Proteobacteria. The purple nodes represent viruses, while the green nodes indicate bacteria. In general, a virus has at least one host. The number of edges means the number of hosts for this virus, while the color of the edges represents the likelihood that the bacterium is the host of the virus. The darker the edge color, the higher the proportion that the virus parasitizes in this bacterium.

**Fig. S11. Distribution of ARG-associated HGT events in water, sediment, and crayfish gut habitats.** (A) The abundance of ARGs Types (TPM) that were involved in HGT events. (B) The ratio of ARGs that were involved in HGT events. (C) The proportion of ARGs’ hosts detected from the bacterial contigs in the water habitat. (D) The proportion of ARGs’ host detected from the bacterial contigs in crayfish gut habitat.

**Fig. S12. ARG-associated HGT events across kingdoms in sediment habitat.** Only four ARG-associated HGT events were detected in non-RCFP models, no ARG-associated HGT events were detected in RCFP model. Each band in the inner or outer circle represents a microbe at the genus level, and the name of microbes is colored according to the kingdom type: bacteria (black) and archaea (red). Bands among bacterial and archaeal genomes mean the ARGs are involved in HGT events across kingdoms. Different band colors represent different microbes bearing ARGs involved in HGT events.

**Fig. S13. MetaCHIP predicted HGT events among kingdoms and aquaculture models in water habitat.** HGT events were detected in (A) RCFP and (B) non-RCFP models, respectively. Each circle band represents a microbe at the genus level, and the one color of a circle band represents a microbe. Bands among the genomes of microbes mean the genes are involved in HGT events across kingdoms. Different band colors represent different microbes bearing functional genes involved in HGT events.

**Fig. S14. MetaCHIP predicted HGT events among kingdoms in crayfish gut habitat.** Through MetaCHIP analysis, only 4 HGT events were detected in non-RCFP models (on average 0.8 HGT events per sample). Each circle band represents a microbe at the genus level, and the one color of a circle band represents a microbe. Bands among the genomes of microbes mean the genes are involved in HGT events across kingdoms. Different band colors represent different microbes bearing functional genes involved in HGT events.

**Supplementary Table captions**

**Table S1**. **Distribution of successfully sequenced samples collected from four representative aquaculture models at Honghu, Hubei province, China.** Note that “0” means we collected the sample but it could not be sequenced. “1” means we collected one sample in this model and this sample was successfully sequenced. “2” means we collected two samples in this model and these two samples were successfully sequenced.

**Table S2. The reads number, base-pairs, and the average length of reads in raw sequencing data, clean data, and the assembled contigs data in each sample.**

**Table S3. The proportion of reads that could be assigned to different taxonomic levels in water, sediment, crayfish gut, and crab gut habitats, respectively.**

**Table S4. The minimum and maximum number of microbial taxa obtained within a habitat.**

**Table S5. The detailed information of indicator microbes for microbial community across aquaculture models in water habitat.** Only the microbes of an aquaculture model with the highest indicator values at p < 0.05 were considered indicator microbes for this model. Except for viruses (family level), all indicator microbes were detected at the genus level.

**Table S6. The detailed information of indicator microbes for microbial community across aquaculture models in sediment habitat.** Only the microbes of an aquaculture model with the highest indicator values at p < 0.05 were considered indicator microbes for this model. Except for viruses (family level), all indicator microbes were detected at the genus level.

**Table S7. The detailed information of indicator microbes for microbial community across aquaculture models in crayfish gut habitat.** Only the microbes of an aquaculture model with the highest indicator values at p < 0.05 were considered indicator microbes for this model. Except for viruses (family level), all indicator microbes were detected at the genus level.

**Table S8. The detailed information for indicator microbes of microbial community across aquaculture models in crab gut habitat.** Only the microbes of an aquaculture model with the highest indicator values at p < 0.05 were considered indicator microbes for this model. Except for viruses (family level), all indicator microbes were detected at the genus level.

**Table S9. Network properties for RCFP and non-RCFP in water, sediment, and crayfish gut habitats, respectively.**

**Supplementary Figures**


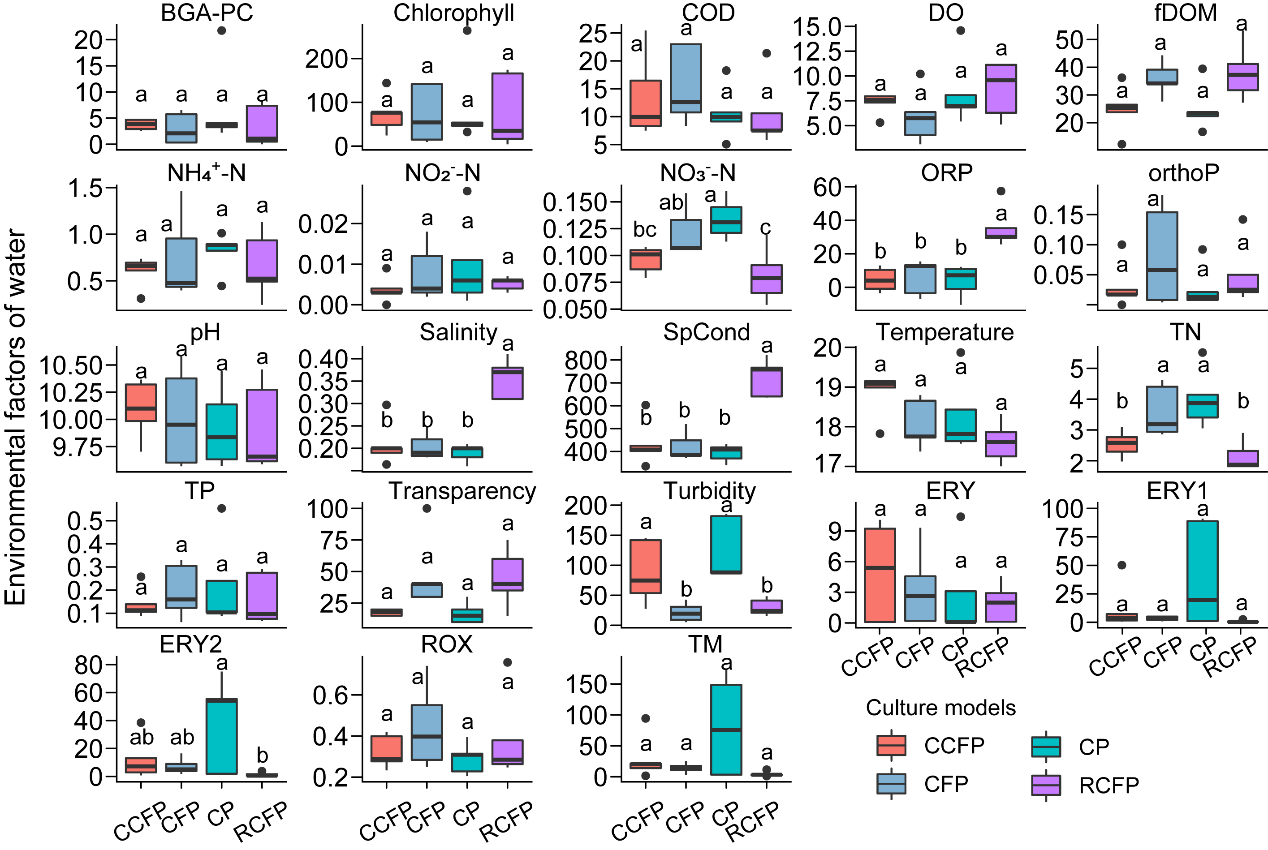


**Fig. S1. Comparison of water environmental factors among different aquaculture models.** The variation among different aquaculture models was tested by variance with the least-significant difference tests. The presence of different letters denoted significant differences (p < 0.05) among culture models, and the same letter indicated no significant difference. BGA-PC: blue-green alga phycocyanin chlorophyll; COD: chemical oxygen demand; DO: dissolved oxygen; fDOM: fluorescent dissolved organic matter; NH_4_^+^-N: ammonium nitrogen concentration; NO_2_^-^-N: nitrite-nitrogen concentration; NO_3_^−^-N: nitrate-nitrogen concentration; ORP: oxidation-reduction potential; orthoP: orthophosphate; SpCond: specific conductance; TN: total nitrogen; TP: total phosphorus; ERY: Erythromycin; ERY1: erythromycin derivative 1; ERY2: erythromycin derivative 2; ROX: roxithromycin; TM: Total macrolide.


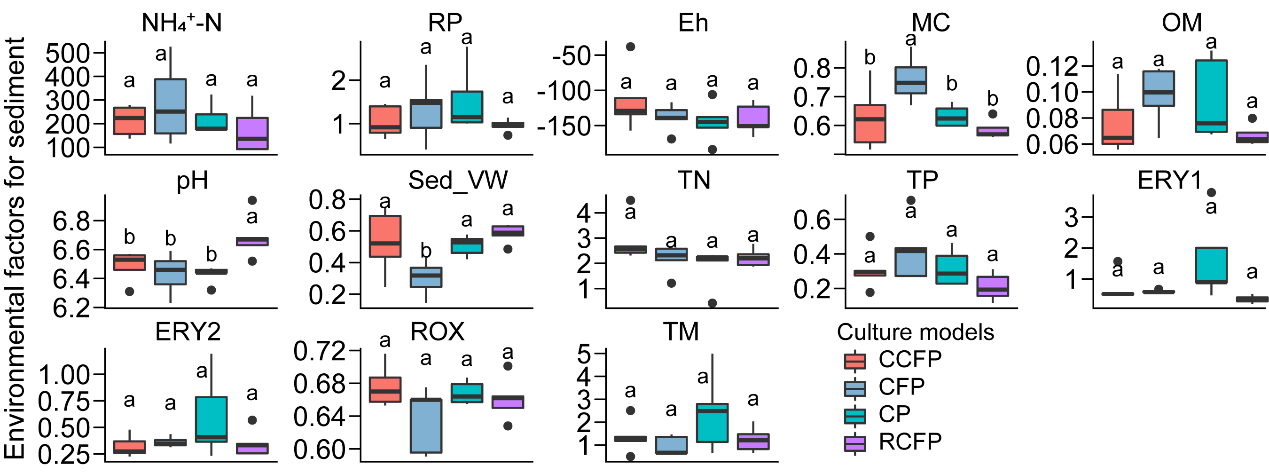


**Fig. S2. Comparison of sediment environmental factors among different aquaculture models.** The variation among different types of culture models was tested by variance with the least-significant difference tests. The presence of different letters denoted significant differences (p < 0.05) among culture models and the same letter indicated no significant difference. NH_4_^+^-N: ammonium nitrogen concentration; RP: released phosphorus concentration; Eh: electric potential half cells; MC: moisture content; OM: proportion of organic matter; Sed_VM: volume-weight of sediment; TN: total nitrogen; TP: total phosphorus; ERY1: erythromycin derivative 1; ERY2: erythromycin derivative 2; ROX: roxithromycin; TM: Total macrolide.

**
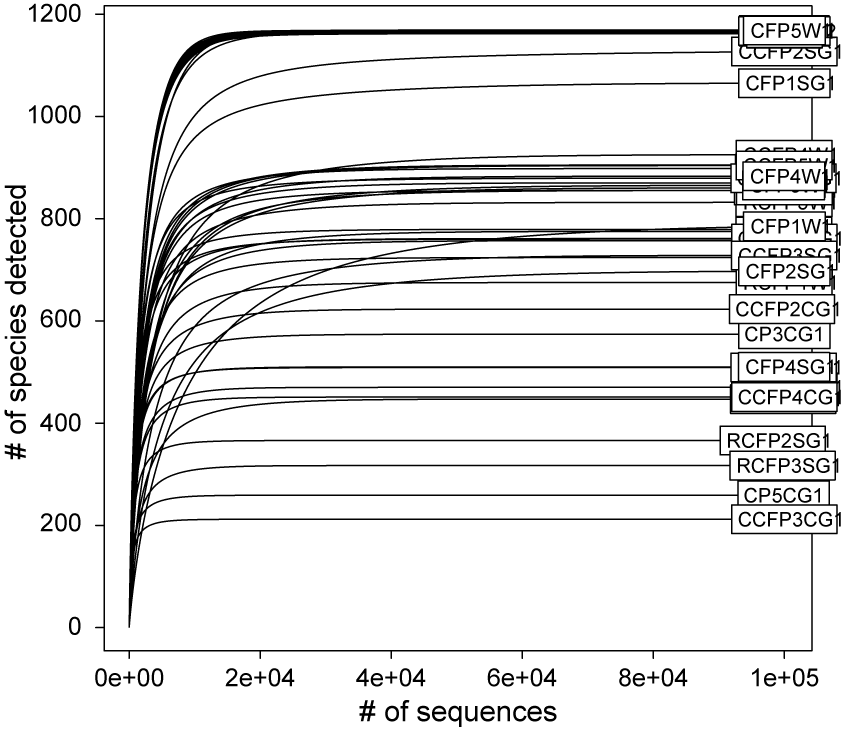
**

**Fig. S3**. **Rarefaction carves of sequencing depth of 58 successfully sequenced samples collected from Honghu, Hubei province, China.** Each curve represents a sample. The x-axis represents the number of sequences, and the y-axis represents the number of detected species.


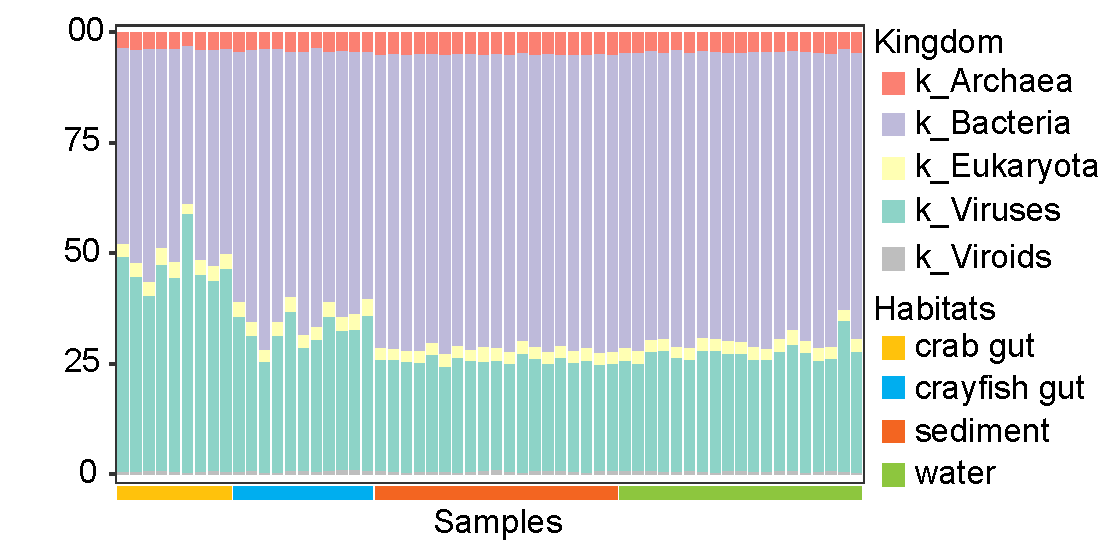


**Fig. S4. Microbial composition at kingdom level.** The horizontal bar colored in yellow, blue, orange, and green color represented the samples collected from crab gut, crayfish gut, sediment, and water habitat, respectively. Each vertical column represented a sample. Each color in a vertical column represents a kingdom.


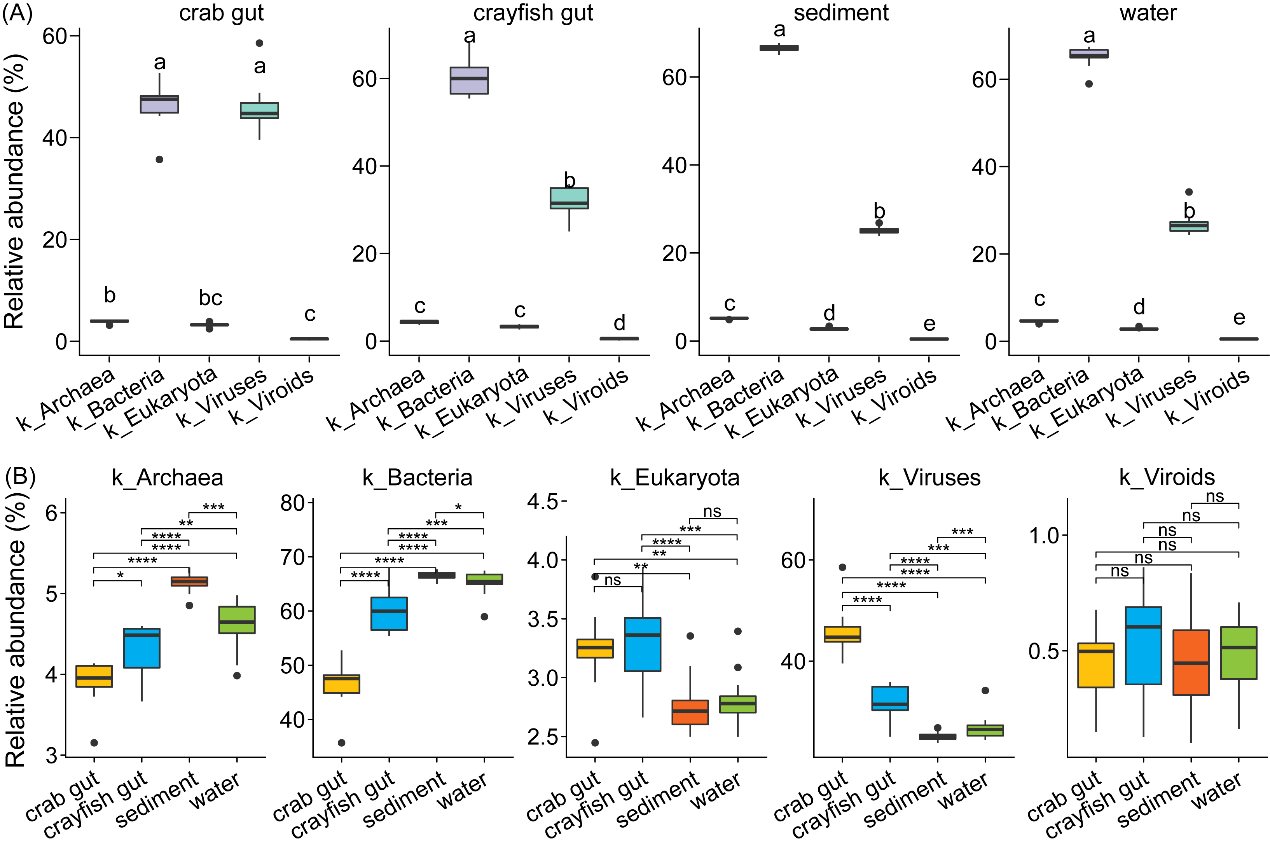


**Fig. S5. Comparison of the relative abundance of kingdoms within and among habitats.** (A) Comparison of the relative abundance of Archaea, Bacteria, Eukaryota, Viruses, and Viroids to determine the dominated kingdom within a habitat. (B) Comparison of the relative abundance of a kingdom among carb gut, crayfish gut, sediment, and water habitat to detect the dominated kingdom among habitats. The variation among culture models was tested by variance with the least-significant difference tests. The presence of different letters denoted significant differences (p < 0.05) among culture models and the same letter indicated no significant difference.


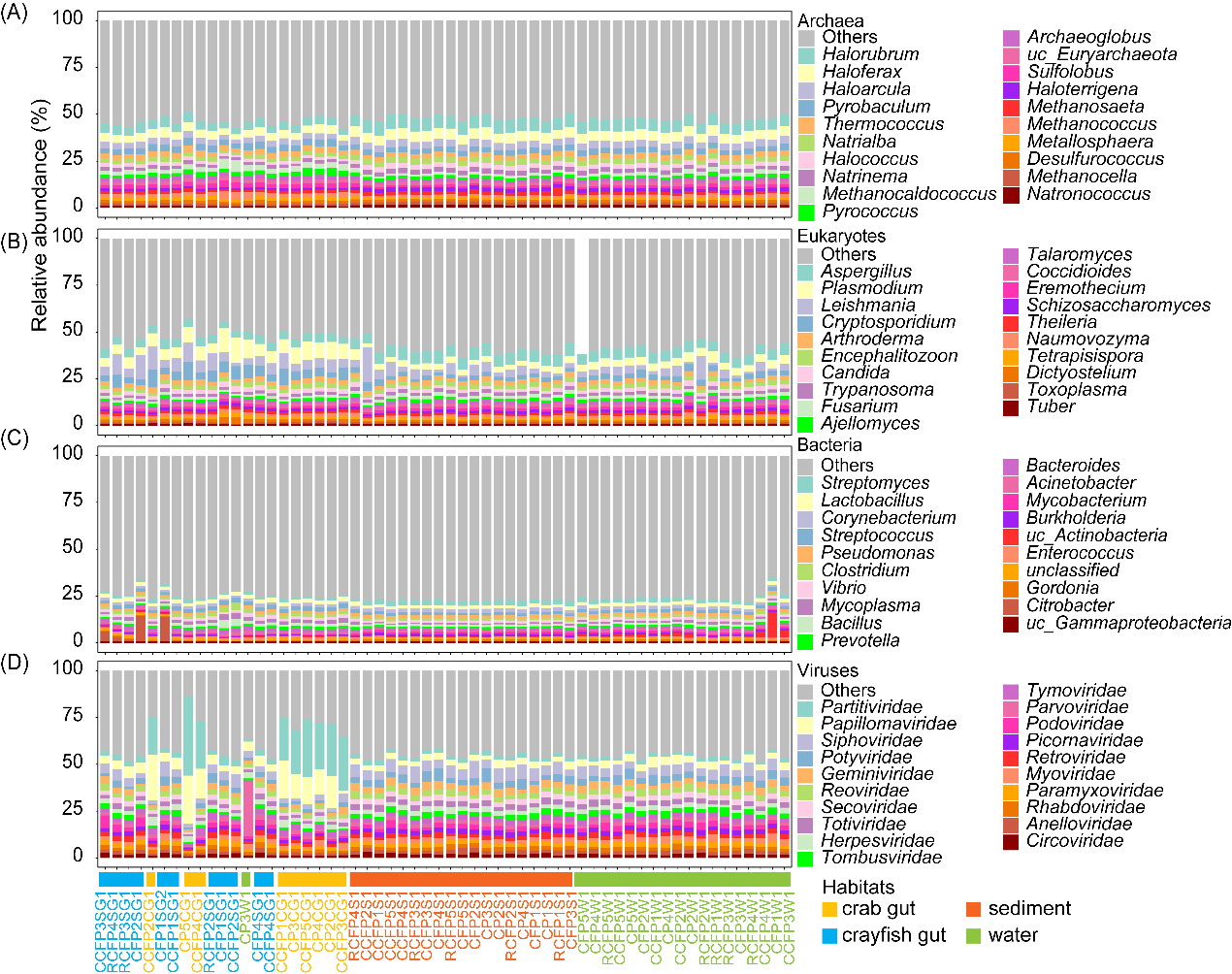


**Fig. S6. Microbial composition among kingdoms.** (A) Archaea community. (B) Eukaryotic community. (C) Viral community. For archaea and eukaryotic composition were based on the top 20 genera. The horizontal bar colored in yellow, blue, orange and green color represented the samples collected from crab gut, crayfish gut, sediment, and water habitats, respectively. Each vertical column represented a sample. Others mean the microbes were not among the top 20 microbes. “uc’’: unclassified.


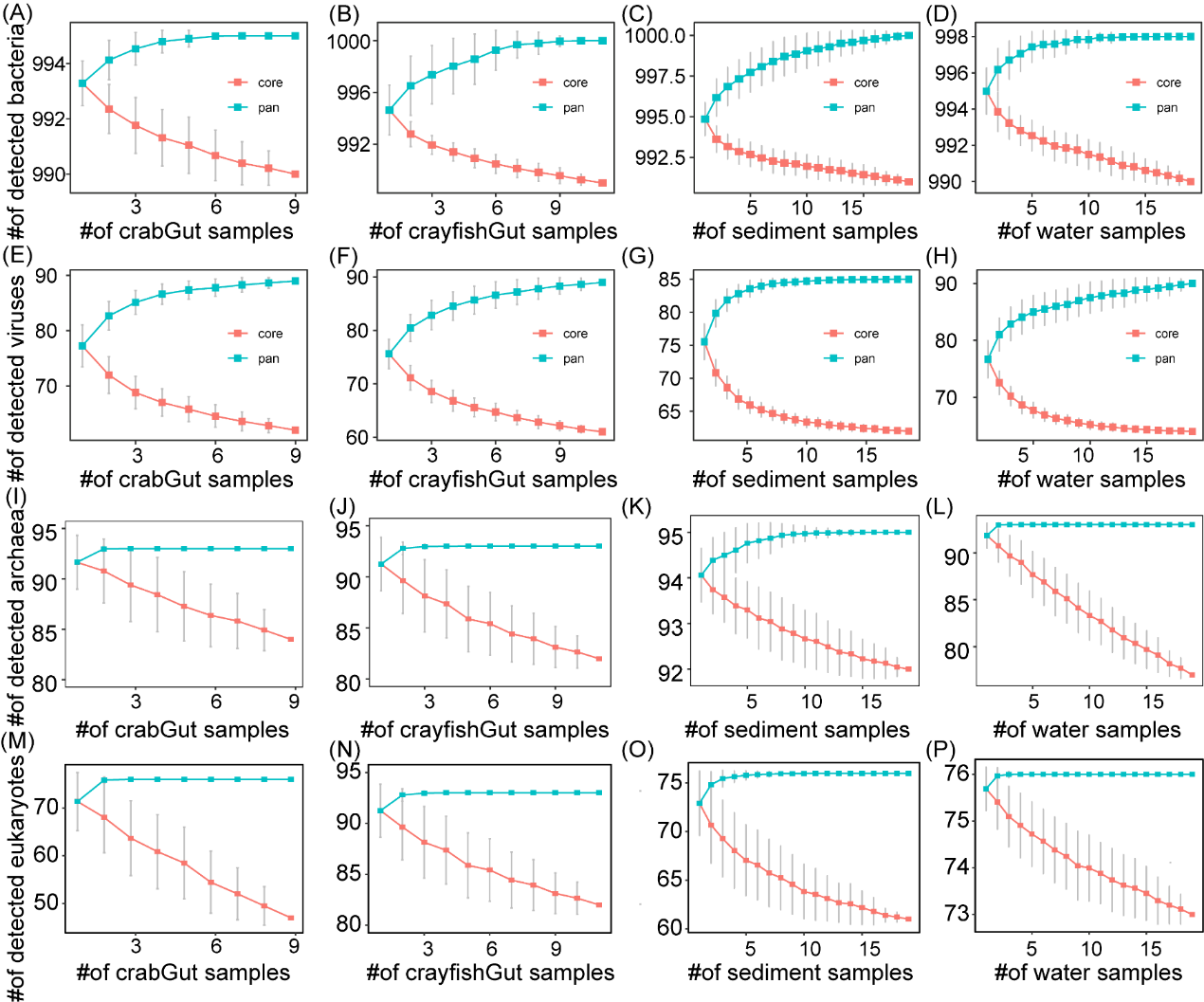


**Fig. S7. Core- and pan-taxa curves within each habitat.** The pan (the blue curve) and the core bacteria (the red curve) for crab gut samples (A), crayfish gut samples (B), sediment samples (C), and water samples (D), respectively. The pan (the blue curve) and the core viruses (the red curve) for crab gut samples (E), crayfish gut samples (F), sediment samples (G), and water samples (H), respectively. The pan (the blue curve) and the core archaea (the red curve) for crab gut samples (I), crayfish gut samples (J), sediment samples (K), and water samples (L), respectively. The pan (the blue curve) and the core eukaryotes (the red curve) for crab gut samples (M), crayfish gut samples (N), sediment samples (O), and water samples (P), respectively. The x-axis represents the number of samples, while y-axis means the number of detected core or pan taxa when adding a sample.


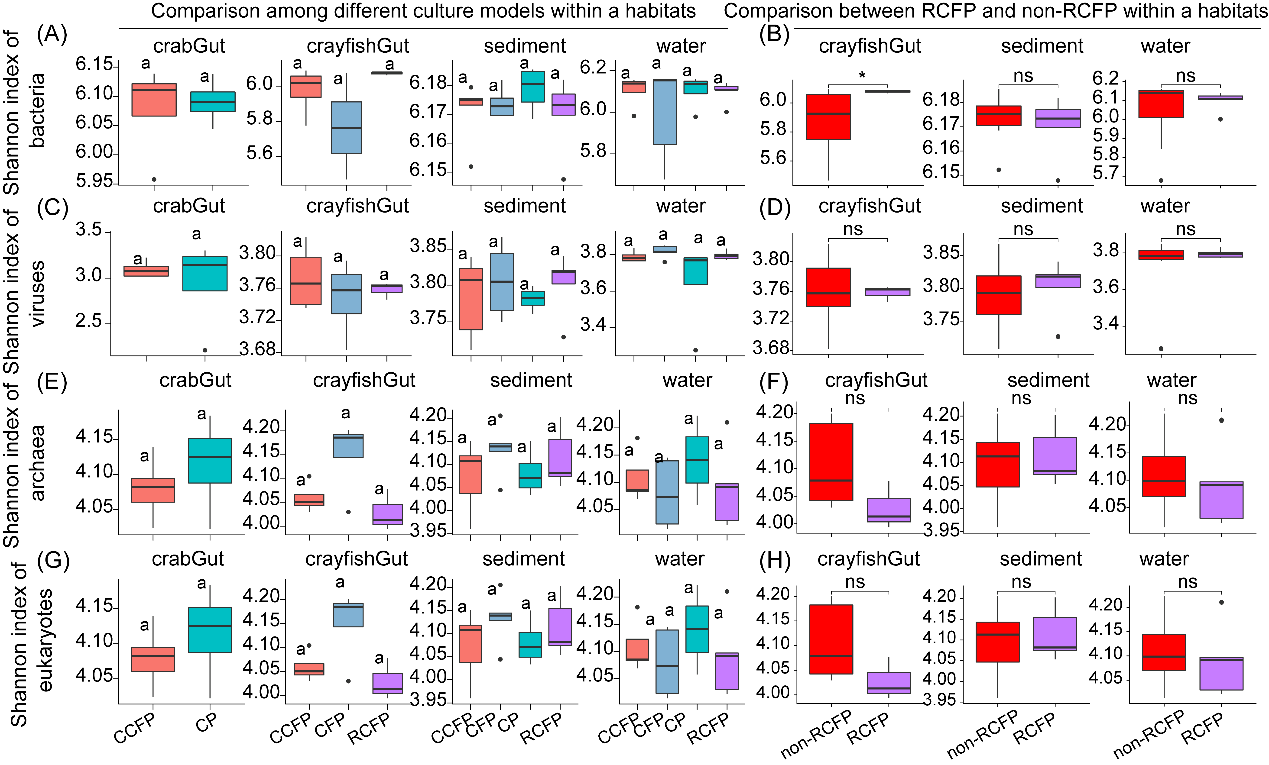


**Fig. S8. Comparison of microbial diversity among different types of culture models.** (A) Comparison of bacterial diversity among different culture models within each habitat. (B) Comparison of bacterial diversity between RCFP and non-RCFP within each habitat. (C) Comparison of viral diversity among crab gut, crayfish gut, water, and sediment samples based on Shannon index. (D) Comparison of viral diversity among different culture models within each habitat. (E) Comparison of archaea diversity among different culture models within each habitat. (F) Comparison of the archaea diversity between RCFP and non-RCFP within each habitat. (G) Comparison of eukaryotic diversity among different culture models within each habitat. (H) Comparison of eukaryotic diversity between RCFP and non-RCFP within each habitat. Water: colored in green; Sediment: colored in orange; crab gut: colored in yellow; crayfish gut: colored in blue. CCFP: crab-crayfish culture model; CFP: crayfish culture model; CP: crab culture model; RCFP: rice-crayfish culture model. The variations among culture models were tested through an analysis of variance with least-significant-difference tests. The presence of different letters denoted significant differences (p < 0.05) among culture models, and the same letter indicated no significant difference.


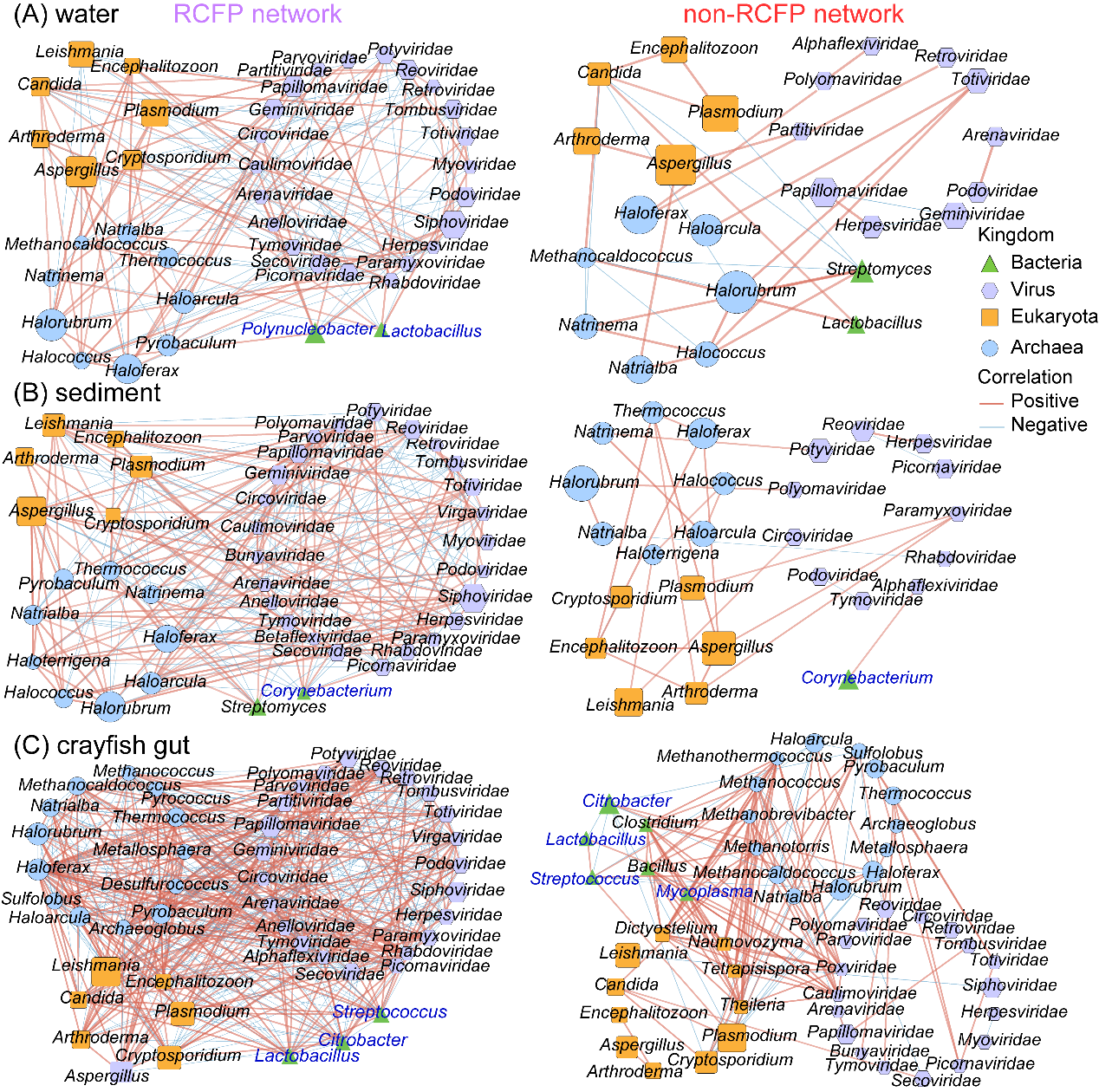


**Fig. S9. Multi-kingdom co-occurrence networks of samples in RCFP and non-RCFP groups with all microbe names annotated.** Networks in (A) water, (B) sediment, and (C) crayfish gut habitats are presented, respectively. Microbes with a relative abundance of ≥2% and coverage of >20% samples were used to construct the multi-kingdom co-occurrence networks. Only Spearman correlations of ≥0.65 or ≤−0.65 with p < 0.05 were considered strong correlations and visualized in the network. The nodes in green, blue, purple, and orange represent bacteria, archaea, viruses, and eukaryotes, respectively. Red edges indicate positive correlations, whereas blue edges reflect negative correlations.

**
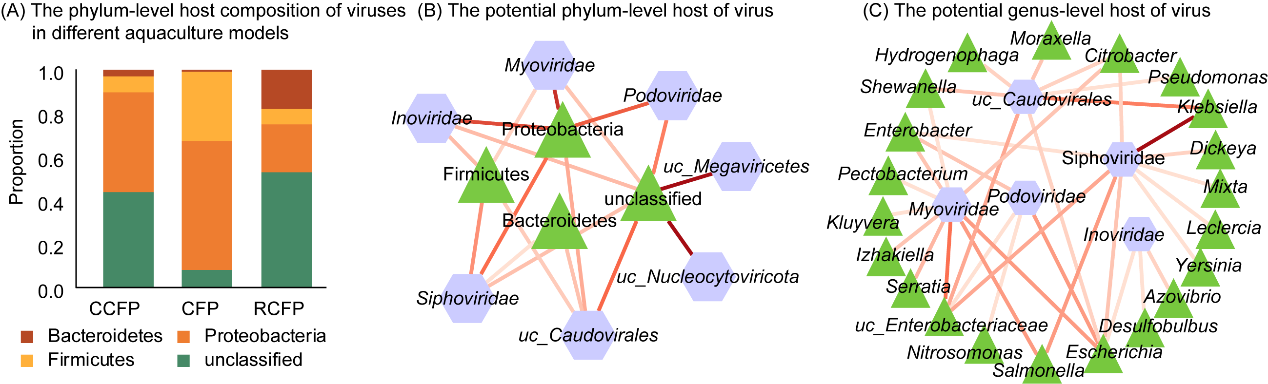
**

**Fig. S10. The host distribution of viruses by querying the IMG/VR and RefSeq databases in crayfish gut habitat.** (A) The phylum-level bacterial host of virus in different aquaculture models by querying the IMG/VR and RefSeq database. (B) The potential phylum-level bacterial host of viruses. (C) The potential genus-level host of the virus. The genus-level hosts of viruses were from Proteobacteria. The purple nodes represent viruses, while the green nodes indicate bacteria. In general, a virus has at least one host. The number of edges means the number of hosts for this virus, while the color of the edges represents the likelihood that the bacterium is the host of the virus. The darker the edge color, the higher the proportion that the virus parasitizes in this bacterium.


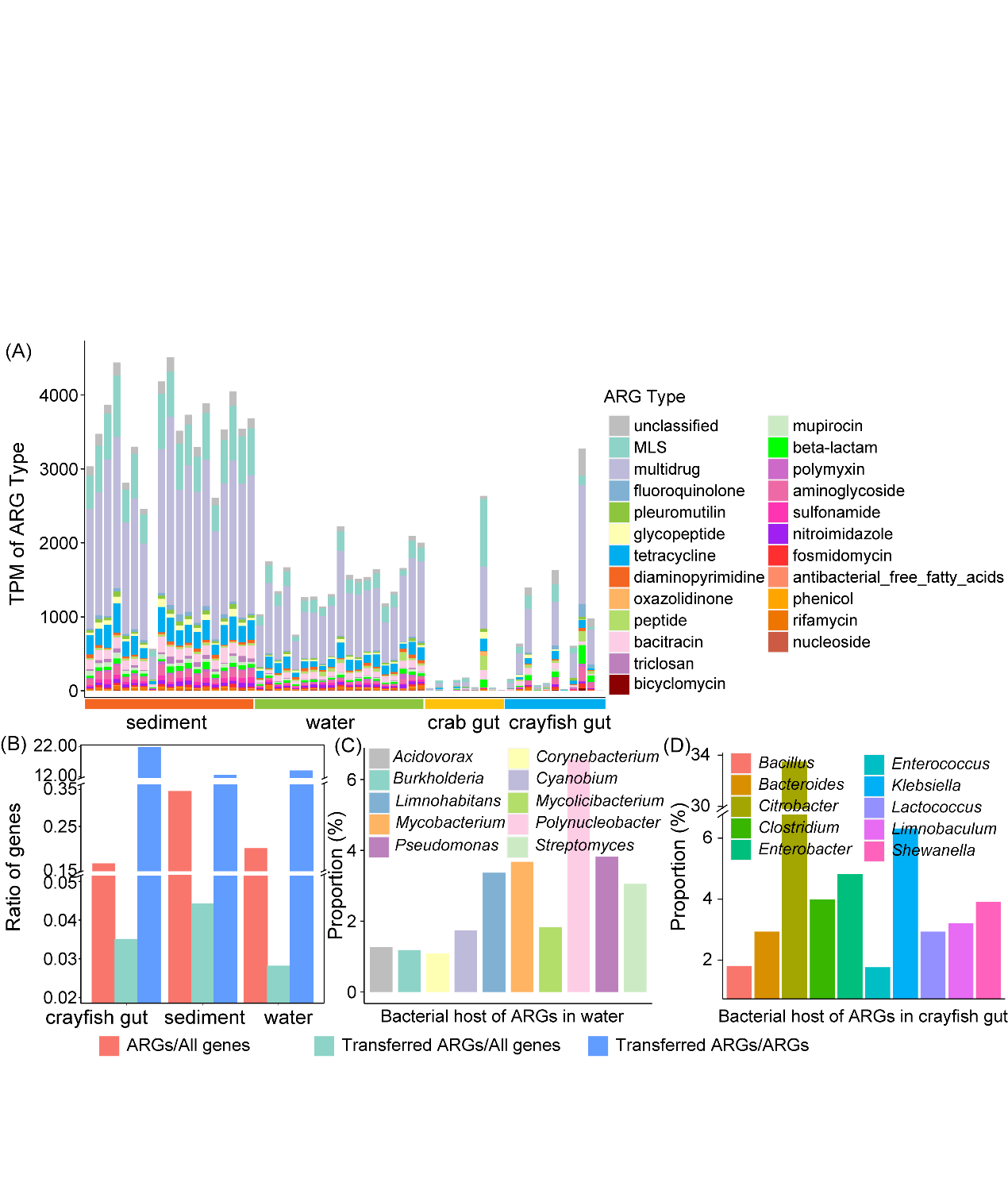


**Fig. S11. Distribution of ARG-associated HGT events in water, sediment, and crayfish gut habitats.** (A) The abundance of ARGs Types (TPM) that were involved in HGT events. (B) The ratio of ARGs that were involved in HGT events. (C) The proportion of ARGs’ hosts detected from the bacterial contigs in the water habitat. (D) The proportion of ARGs’ host detected from the bacterial contigs in crayfish gut habitat.


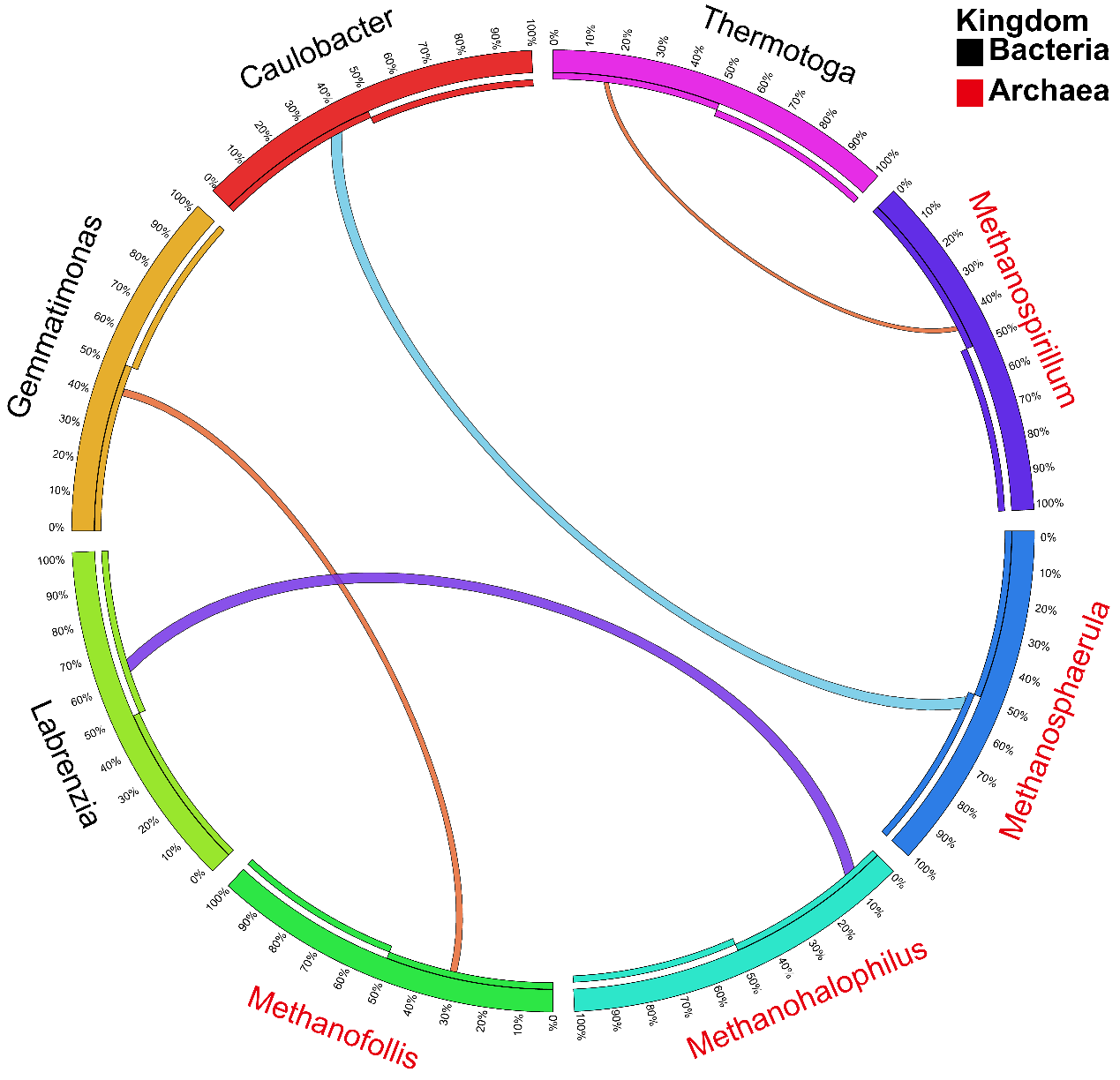


**Fig. S12.** **ARG-associated HGT events across kingdoms in sediment habitat.** Only four ARG-associated HGT events were detected in non-RCFP models, no ARG-associated HGT events were detected in RCFP model. Each band in the inner or outer circle represents a microbe at the genus level, and the name of microbes is colored according to the kingdom type: bacteria (black) and archaea (red). Bands among bacterial and archaeal genomes mean the ARGs are involved in HGT events across kingdoms. Different band colors represent different microbes bearing ARGs involved in HGT events.


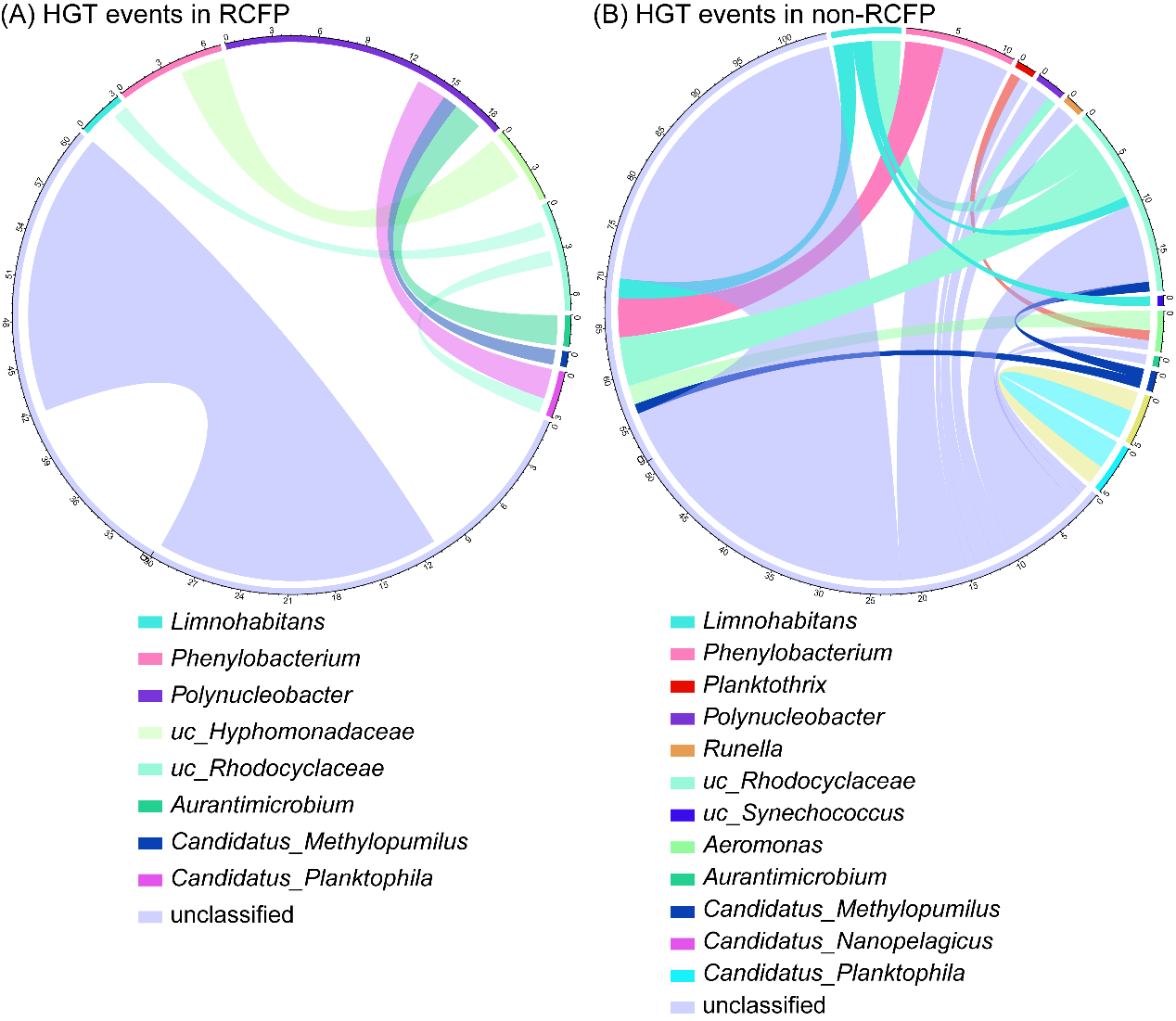


**Fig. S13. MetaCHIP predicted HGT events among kingdoms and aquaculture models in water habitat.** HGT events were detected in (A) RCFP and (B) non-RCFP models, respectively. Each circle band represents a microbe at the genus level, and the one color of a circle band represents a microbe. Bands among the genomes of microbes mean the genes are involved in HGT events across kingdoms. Different band colors represent different microbes bearing functional genes involved in HGT events.

**
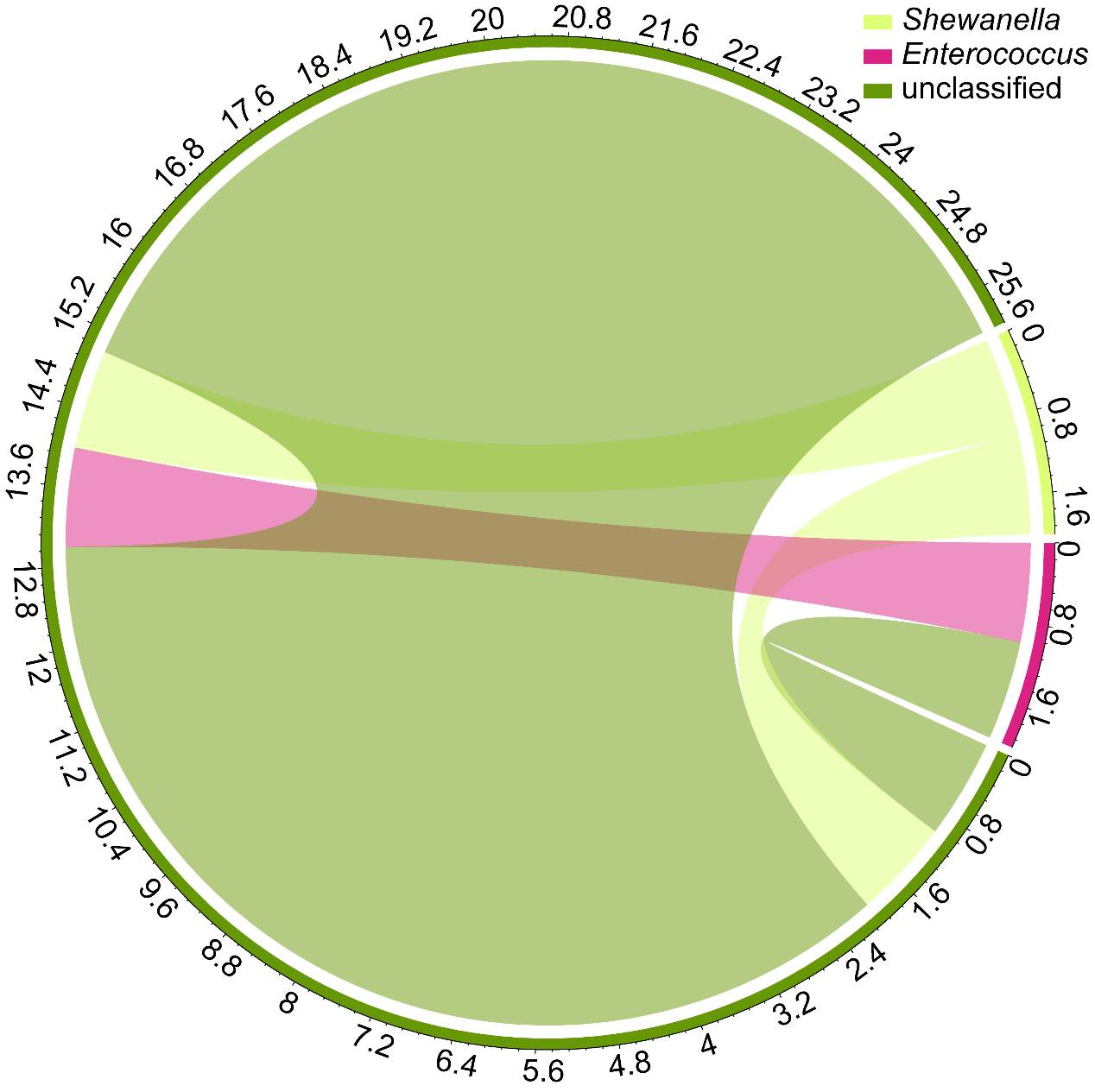
**

**Fig. S14. MetaCHIP predicted HGT events among kingdoms in crayfish gut habitat.** Through MetaCHIP analysis, only 4 HGT events were detected in non-RCFP models (on average 0.8 HGT events per sample). Each circle band represents a microbe at the genus level, and the one color of a circle band represents a microbe. Bands among the genomes of microbes mean the genes are involved in HGT events across kingdoms. Different band colors represent different microbes bearing functional genes involved in HGT events.

**Supplementary Tables**

**Table S1**. **Distribution of successfully sequenced samples collected from four representative aquaculture models at Honghu, Hubei province, China.**

| Aquaculture models | Culture models | Water (19 samples) | Sediment  (19 samples) | Crab gut (9 samples) | Crayfish gut (11 samples) |
| --- | --- | --- | --- | --- | --- |
| Crab culture model (CP) 12 samples | P1 | 0 | 1 | 0 | 0 |
|  | P2 | 1 | 1 | 1 | 0 |
|  | P3 | 1 | 1 | 1 | 0 |
|  | P4 | 1 | 1 | 1 | 0 |
|  | P5 | 1 | 0 | 1 | 0 |
| Crayfish culture model (CFP) 14 samples | P1 | 1 | 1 | 0 | 2 |
|  | P2 | 1 | 1 | 0 | 1 |
|  | P3 | 1 | 1 | 0 | 0 |
|  | P4 | 1 | 1 | 0 | 1 |
|  | P5 | 1 | 1 | 0 | 0 |
| Crayfish-crab culture model (CCFP) 19 samples | P1 | 1 | 1 | 1 | 1 |
|  | P2 | 1 | 1 | 1 | 1 |
|  | P3 | 1 | 1 | 1 | 1 |
|  | P4 | 1 | 1 | 1 | 1 |
|  | P5 | 1 | 1 | 1 | 0 |
| Rice-crayfish culture model (RCFP) 13 samples | P1 | 1 | 1 | 0 | 0 |
|  | P2 | 1 | 1 | 0 | 1 |
|  | P3 | 1 | 1 | 0 | 1 |
|  | P4 | 1 | 1 | 0 | 1 |
|  | P5 | 1 | 1 | 0 | 0 |

Note that “0” means we collected the sample but it could not be sequenced. “1” means we collected one sample in this model and this sample was successfully sequenced. “2” means we collected two samples in this model and these two samples were successfully sequenced.

**Table S2. The** **reads number, base-pairs, and the average length of reads in raw sequencing data, clean data, and the assembled contigs data in each sample.**

| SampleID | Raw data | | | Clean data | | | Contigs | | |
| --- | --- | --- | --- | --- | --- | --- | --- | --- | --- |
|  | Reads number | Base-pairs (bp) | Average length (bp) | Reads number | Base-pairs (bp) | Average length (bp) | Reads number | Base-pairs (bp) | Average length (bp) |
| CP1S1 | 18,441,347 | 2,766,202,050 | 150 | 18,099,446 | 2,563,631,684 | 141.64 | 13,315 | 18,534,210 | 1,391.98 |
| CP2CG1 | 21,595,772 | 3,239,365,800 | 150 | 20,935,243 | 2,716,176,788 | 129.74 | 5,299 | 7,604,760 | 1,435.13 |
| CP2S1 | 22,233,675 | 3,335,051,250 | 150 | 21,868,592 | 3,083,496,775 | 141.00 | 21,908 | 31,590,904 | 1,441.98 |
| CP2W1 | 19,052,646 | 2,857,896,900 | 150 | 18,647,122 | 2,608,469,118 | 139.89 | 86,519 | 154,444,015 | 1,785.09 |
| CP3CG1 | 19,470,732 | 2,920,609,800 | 150 | 18,972,551 | 2,482,415,889 | 130.84 | 10,380 | 20,519,850 | 1,976.86 |
| CP3S1 | 22,840,782 | 3,426,117,300 | 150 | 22,476,750 | 3,172,799,320 | 141.16 | 12,181 | 17,283,829 | 1,418.92 |
| CP3W1 | 18,126,501 | 2,718,975,150 | 150 | 15,631,900 | 1,814,176,167 | 116.06 | 29,726 | 48,259,163 | 1,623.47 |
| CP4CG1 | 20,988,407 | 3,148,261,050 | 150 | 20,281,257 | 2,632,975,208 | 129.82 | 10,583 | 13,444,341 | 1,270.37 |
| CP4S1 | 23,620,433 | 3,543,064,950 | 150 | 23,241,456 | 3,276,837,529 | 140.99 | 13,262 | 19,363,953 | 1,460.11 |
| CP4W1 | 22,239,971 | 3,335,995,650 | 150 | 21,865,345 | 3,076,405,223 | 140.70 | 108,284 | 214,368,958 | 1,979.69 |
| CP5CG1 | 18,096,333 | 2,714,449,950 | 150 | 15,211,532 | 1,888,058,162 | 124.12 | 10,385 | 18,361,260 | 1,768.06 |
| CP5W1 | 23,318,822 | 3,497,823,300 | 150 | 22,949,699 | 3,220,475,792 | 140.33 | 115,319 | 222,994,688 | 1,933.72 |
| RCFP1S1 | 22,525,757 | 3,378,863,550 | 150 | 22,141,092 | 3,121,131,927 | 140.97 | 19,022 | 29,363,504 | 1,543.66 |
| RCFP1W1 | 21,543,926 | 3,231,588,900 | 150 | 21,189,837 | 2,993,068,217 | 141.25 | 85,830 | 178,201,973 | 2,076.22 |
| RCFP2S1 | 22,409,646 | 3,361,446,900 | 150 | 22,047,937 | 3,115,119,988 | 141.29 | 18,473 | 26,262,493 | 1,421.67 |
| RCFP2SG1 | 17,098,299 | 2,564,744,850 | 150 | 16,563,422 | 2,185,670,554 | 131.96 | 4,675 | 10,204,011 | 2,182.68 |
| RCFP2W1 | 20,360,542 | 3,054,081,300 | 150 | 19,934,178 | 2,762,636,613 | 138.59 | 66,411 | 128,599,328 | 1,936.42 |
| RCFP3S1 | 22,747,061 | 3,412,059,150 | 150 | 22,346,582 | 3,137,262,845 | 140.39 | 14,024 | 22,262,305 | 1,587.44 |
| RCFP3SG1 | 18,306,001 | 2,745,900,150 | 150 | 17,766,917 | 2,358,970,476 | 132.77 | 7,942 | 26,561,276 | 3,344.41 |
| RCFP3W1 | 23,125,130 | 3,468,769,500 | 150 | 22,798,746 | 3,226,038,364 | 141.50 | 106,719 | 224,562,563 | 2,104.24 |
| RCFP4S1 | 20,452,493 | 3,067,873,950 | 150 | 20,059,384 | 2,816,248,946 | 140.40 | 56,258 | 79,227,174 | 1,408.28 |
| RCFP4SG1 | 25,034,015 | 3,755,102,250 | 150 | 21,215,368 | 2,818,633,005 | 132.86 | 6,332 | 15,661,545 | 2,473.40 |
| RCFP4W1 | 22,934,855 | 3,440,228,250 | 150 | 22,589,207 | 3,188,811,132 | 141.17 | 112,167 | 237,191,795 | 2,114.63 |
| RCFP5S1 | 20,353,033 | 3,052,954,950 | 150 | 19,970,820 | 2,822,785,723 | 141.35 | 11,632 | 15,976,781 | 1,373.52 |
| RCFP5W1 | 19,801,608 | 2,970,241,200 | 150 | 19,412,038 | 2,713,127,245 | 139.77 | 73,526 | 148,456,233 | 2,019.10 |
| CCFP1CG1 | 18,974,060 | 2,846,109,000 | 150 | 16,084,207 | 2,064,587,677 | 128.36 | 23,601 | 47,312,941 | 2,004.70 |
| CCFP1S1 | 31,005,877 | 4,650,881,550 | 150 | 26,160,286 | 3,503,993,142 | 133.94 | 8,151 | 11,230,134 | 1,377.76 |
| CCFP1SG1 | 19,553,333 | 2,932,999,950 | 150 | 18,931,476 | 2,508,158,139 | 132.49 | 17,531 | 32,911,423 | 1,877.33 |
| CCFP1W1 | 23,001,202 | 3,450,180,300 | 150 | 22,629,961 | 3,172,883,050 | 140.21 | 121,223 | 250,051,699 | 2,062.74 |
| CCFP2CG1 | 23,069,463 | 3,460,419,450 | 150 | 19,912,395 | 2,520,163,786 | 126.56 | 35,963 | 54,288,386 | 1,509.56 |
| CCFP2S1 | 25,925,756 | 3,888,863,400 | 150 | 21,982,969 | 2,937,065,862 | 133.61 | 11,980 | 16,866,638 | 1,407.90 |
| CCFP2SG1 | 19,317,453 | 2,897,617,950 | 150 | 16,887,857 | 1,504,665,610 | 89.10 | 1,086 | 1,563,340 | 1,439.54 |
| CCFP2W1 | 22,365,435 | 3,354,815,250 | 150 | 21,979,472 | 3,072,707,223 | 139.80 | 89,442 | 175,820,828 | 1,965.75 |
| CCFP3CG1 | 21,162,917 | 3,174,437,550 | 150 | 17,658,896 | 2,313,039,681 | 130.98 | 17,311 | 56,976,022 | 3,291.32 |
| CCFP3S1 | 22,850,878 | 3,427,631,700 | 150 | 22,413,289 | 3,166,839,259 | 141.29 | 13,649 | 18,883,955 | 1,383.54 |
| CCFP3SG1 | 22,253,779 | 3,338,066,850 | 150 | 21,733,901 | 2,992,205,762 | 137.68 | 26,302 | 102,179,037 | 3,884.84 |
| CCFP3W1 | 23,352,538 | 3,502,880,700 | 150 | 22,967,363 | 3,230,695,760 | 140.67 | 105,313 | 206,581,279 | 1,961.59 |
| CCFP4CG1 | 17,261,513 | 2,589,226,950 | 150 | 16,832,826 | 2,210,816,571 | 131.34 | 44,870 | 67,449,776 | 1,503.23 |
| CCFP4S1 | 23,496,825 | 3,524,523,750 | 150 | 23,049,544 | 3,248,646,267 | 140.94 | 33,253 | 50,548,552 | 1,520.12 |
| CCFP4SG1 | 21,804,139 | 3,270,620,850 | 150 | 21,177,847 | 2,815,548,057 | 132.95 | 21,837 | 32,998,756 | 1,511.14 |
| CCFP4W1 | 23,732,988 | 3,559,948,200 | 150 | 23,352,695 | 3,271,539,017 | 140.09 | 99,932 | 194,292,373 | 1,944.25 |
| CCFP5CG1 | 23,683,899 | 3,552,584,850 | 150 | 22,971,121 | 2,957,035,750 | 128.73 | 37,041 | 47,784,321 | 1,290.04 |
| CCFP5S1 | 22,224,795 | 3,333,719,250 | 150 | 21,871,995 | 3,086,213,608 | 141.10 | 9,181 | 12,275,440 | 1,337.05 |
| CCFP5W1 | 22,549,431 | 3,382,414,650 | 150 | 22,190,892 | 3,125,121,518 | 140.83 | 122,062 | 228,162,209 | 1,869.23 |
| CFP1S1 | 21,094,682 | 3,164,202,300 | 150 | 20,617,139 | 2,900,680,025 | 140.69 | 18,491 | 26,832,686 | 1,451.12 |
| CFP1SG1 | 17,587,904 | 2,638,185,600 | 150 | 14,362,904 | 1,508,514,616 | 105.03 | 2,695 | 4,411,890 | 1,637.06 |
| CFP1SG2 | 18,045,549 | 2,706,832,350 | 150 | 17,600,080 | 2,404,504,194 | 136.62 | 9,542 | 37,449,043 | 3,924.65 |
| CFP1W1 | 22,590,774 | 3,388,616,100 | 150 | 22,233,992 | 3,134,864,872 | 140.99 | 99,552 | 215,300,102 | 2,162.69 |
| CFP2S1 | 22,658,047 | 3,398,707,050 | 150 | 22,313,074 | 3,155,853,151 | 141.44 | 15,809 | 22,674,731 | 1,434.29 |
| CFP2SG1 | 21,326,679 | 3,199,001,850 | 150 | 20,685,878 | 2,756,739,472 | 133.27 | 18,028 | 44,731,327 | 2,481.21 |
| CFP2W1 | 23,227,456 | 3,484,118,400 | 150 | 22,788,193 | 3,201,651,427 | 140.50 | 95,026 | 171,933,812 | 1,809.33 |
| CFP3S1 | 22,688,572 | 3,403,285,800 | 150 | 22,290,134 | 3,153,642,996 | 141.48 | 13,374 | 18,264,867 | 1,365.70 |
| CFP3W1 | 23,099,030 | 3,464,854,500 | 150 | 22,723,965 | 3,205,764,669 | 141.07 | 87,023 | 177,273,030 | 2,037.08 |
| CFP4S1 | 22,811,068 | 3,421,660,200 | 150 | 22,435,952 | 3,167,534,758 | 141.18 | 24,867 | 38,342,324 | 1,541.90 |
| CFP4SG1 | 20,212,989 | 3,031,948,350 | 150 | 19,643,031 | 2,627,987,876 | 133.79 | 1,797 | 2,600,622 | 1,447.20 |
| CFP4W1 | 22,268,982 | 3,340,347,300 | 150 | 21,922,604 | 3,078,714,844 | 140.44 | 123,077 | 234,214,782 | 1,902.99 |
| CFP5S1 | 23,949,909 | 3,592,486,350 | 150 | 23,596,275 | 3,336,126,229 | 141.38 | 32,521 | 52,508,882 | 1,614.61 |
| CFP5W1 | 22,096,914 | 3,314,537,100 | 150 | 21,741,895 | 3,051,104,483 | 140.33 | 115,044 | 214,469,735 | 1,864.24 |

**Table S3. The proportion of reads that could be assigned to different taxonomic levels in water, sediment, crayfish gut, and crab gut habitats, respectively.**

|  | Taxonomic levels | Archaea (%) | Bacteria (%) | Eukaryota (%) | Viruses (%) | Unclassified (%) |
| --- | --- | --- | --- | --- | --- | --- |
| water | Kingdom | 4.62 | 65.41 | 2.79 | 26.70 | 0.48 |
|  | Phylum | 4.59 | 65.27 | 2.37 | -- | 1.06 |
|  | Class | 4.23 | 63.27 | 2.26 | -- | 3.54 |
|  | Order | 4.23 | 60.25 | 2.16 | 8.84 | 24.52 |
|  | Family | 4.23 | 58.95 | 1.89 | 23.83 | 11.10 |
|  | Genus | 4.23 | 57.60 | 1.89 | 20.20 | 16.08 |
|  | Species | 4.19 | 56.85 | 1.89 | 19.62 | 17.45 |
|  | Strain | 3.68 | 41.15 | 1.86 | 19.42 | 33.89 |
| sediment | Kingdom | 5.15 | 66.59 | 2.74 | 25.08 | 0.45 |
|  | Phylum | 5.12 | 66.44 | 2.32 | -- | 1.04 |
|  | Class | 4.80 | 64.69 | 2.22 | -- | 3.21 |
|  | Order | 4.76 | 62.88 | 2.13 | 8.38 | 21.85 |
|  | Family | 4.76 | 61.63 | 1.92 | 22.39 | 9.31 |
|  | Genus | 4.76 | 60.34 | 1.92 | 18.76 | 14.23 |
|  | Species | 4.68 | 59.71 | 1.91 | 18.66 | 15.04 |
|  | Strain | 4.10 | 44.51 | 1.89 | 17.87 | 31.63 |
| crayfish gut | Kingdom | 4.32 | 60.18 | 3.28 | 31.68 | 0.53 |
|  | Phylum | 4.31 | 60.04 | 2.61 | -- | 1.37 |
|  | Class | 3.92 | 58.24 | 2.47 | -- | 3.69 |
|  | Order | 3.90 | 56.43 | 2.35 | 10.26 | 27.05 |
|  | Family | 3.90 | 55.30 | 1.96 | 31.68 | 7.15 |
|  | Genus | 3.90 | 53.76 | 1.96 | 24.85 | 15.52 |
|  | Species | 3.90 | 51.91 | 1.96 | 24.60 | 17.62 |
|  | Strain | 3.43 | 36.80 | 1.94 | 23.40 | 34.42 |
| crab gut | Kingdom | 3.88 | 46.28 | 3.23 | 46.15 | 0.45 |
|  | Phylum | 3.87 | 46.28 | 3.23 | -- | 0.47 |
|  | Class | 3.55 | 44.68 | 2.37 | -- | 3.25 |
|  | Order | 3.77 | 43.26 | 2.27 | 11.45 | 4.55 |
|  | Family | 3.53 | 42.34 | 1.82 | 46.15 | 6.15 |
|  | Genus | 3.53 | 41.14 | 1.82 | 39.33 | 12.77 |
|  | Species | 3.52 | 40.82 | 1.82 | 38.19 | 32.68 |
|  | Strain | 3.12 | 29.59 | 1.79 | 38.19 | 27.31 |

**Table S4. The minimum and maximum number of microbial taxa obtained within a habitat.**

|  | water | |  | sediment | |  | crayfish gut | |  | crayfish gut | |
| --- | --- | --- | --- | --- | --- | --- | --- | --- | --- | --- | --- |
|  | min | max |  | min | max |  | min | max |  | min | max |
| Archaea | 77 | 93 |  | 92 | 95 |  | 84 | 94 |  | 82 | 93 |
| Bacteria | 990 | 1,016 |  | 991 | 1,000 |  | 991 | 1,003 |  | 990 | 1,011 |
| Eukaryotes | 73 | 76 |  | 61 | 76 |  | 47 | 76 |  | 49 | 76 |
| Viruses | 64 | 90 |  | 62 | 85 |  | 62 | 89 |  | 61 | 89 |
| Total | 1,204 | 1,275 |  | 1,206 | 1,256 |  | 1,184 | 1,262 |  | 1,182 | 1,269 |

**Table S5. The detailed information of indicator microbes for microbial community across aquaculture models in water habitat.**

| Kingdom | Indicator microbes | CCFP | CFP | CP | RCFP | p-value (<0.05) | Culture model | Kingdom | Indicator species | CCFP | CFP | CP | RCFP | p-value (<0.05) | Culture model |
| --- | --- | --- | --- | --- | --- | --- | --- | --- | --- | --- | --- | --- | --- | --- | --- |
| Bacteria | *Agarivorans* | 0.2525 | 0.2322 | 0.2199 | 0.2953 | 0.003 | RCFP | Bacteria | *Oscillatoria* | 0.2480 | 0.2410 | 0.2448 | 0.2662 | 0.033 | RCFP |
| Bacteria | *Alloiococcus* | 0.2482 | 0.2443 | 0.2316 | 0.2758 | 0.009 | RCFP | Bacteria | *Parvimonas* | 0.2058 | 0.1910 | 0.3676 | 0.2356 | 0.034 | CP |
| Bacteria | *Alloprevotella* | 0.2572 | 0.2372 | 0.2361 | 0.2695 | 0.027 | RCFP | Bacteria | *Polaribacter* | 0.2065 | 0.2126 | 0.3410 | 0.2400 | 0.025 | CP |
| Bacteria | *Alteromonas* | 0.2064 | 0.1756 | 0.4326 | 0.1854 | 0.007 | CP | Bacteria | *Roseomonas* | 0.3419 | 0.2383 | 0.2112 | 0.2086 | 0.036 | CCFP |
| Bacteria | *Aminobacterium* | 0.2436 | 0.2422 | 0.2324 | 0.2818 | 0.028 | RCFP | Bacteria | *Segetibacter* | 0.2417 | 0.2355 | 0.2453 | 0.2774 | 0.024 | RCFP |
| Bacteria | *Blattabacterium* | 0.1124 | 0.1426 | 0.2599 | 0.4851 | 0.023 | RCFP | Bacteria | *Sphingobacterium* | 0.2208 | 0.2140 | 0.3301 | 0.2351 | 0.037 | CP |
| Bacteria | *Caminibacter* | 0.2248 | 0.2003 | 0.3330 | 0.2419 | 0.012 | CP | Bacteria | *Sulfurihydrogenibium* | 0.2165 | 0.2101 | 0.3316 | 0.2418 | 0.046 | CP |
| Bacteria | *Cellulosilyticum* | 0.2365 | 0.2168 | 0.2908 | 0.2559 | 0.038 | CP | Bacteria | *Thermodesulfatator* | 0.2447 | 0.2391 | 0.2437 | 0.2725 | 0.029 | RCFP |
| Bacteria | *Chitinophaga* | 0.2389 | 0.2450 | 0.2440 | 0.2720 | 0.023 | RCFP | Bacteria | *Thiomicrospira* | 0.2521 | 0.2397 | 0.2392 | 0.2690 | 0.005 | RCFP |
| Bacteria | *Coprococcus* | 0.2452 | 0.2333 | 0.2742 | 0.2474 | 0.039 | CP | Bacteria | *Thiorhodospira* | 0.2557 | 0.2434 | 0.2321 | 0.2689 | 0.003 | RCFP |
| Bacteria | *Cylindrospermum* | 0.2299 | 0.2262 | 0.2405 | 0.3034 | 0.014 | RCFP | Bacteria | *Vagococcus* | 0.2588 | 0.2133 | 0.2415 | 0.2865 | 0.031 | RCFP |
| Bacteria | *Desulfotomaculum* | 0.2525 | 0.2404 | 0.2444 | 0.2627 | 0.041 | RCFP | Bacteria | *Virgibacillus* | 0.2319 | 0.2130 | 0.3204 | 0.2346 | 0.018 | CP |
| Bacteria | *Enhydrobacter* | 0.2317 | 0.2227 | 0.2350 | 0.3106 | 0.029 | RCFP | Bacteria | *Vitreoscilla* | 0.2498 | 0.2269 | 0.2310 | 0.2923 | 0.012 | RCFP |
| Bacteria | *Fibrobacter* | 0.2562 | 0.2403 | 0.2287 | 0.2748 | 0.048 | RCFP | Bacteria | *Xenorhabdus* | 0.2385 | 0.2373 | 0.2490 | 0.2752 | 0.037 | RCFP |
| Bacteria | *Fluoribacter* | 0.2264 | 0.2276 | 0.3121 | 0.2339 | 0.036 | CP | Viruses | *Arenaviridae* | 0.3406 | 0.2086 | 0.1975 | 0.2534 | 0.029 | CCFP |
| Bacteria | *uc_Gammaproteobacteria* | 0.2510 | 0.2422 | 0.2430 | 0.2639 | 0.039 | RCFP | Viruses | *Hepadnaviridae* | 0.0512 | 0.1905 | 0.0129 | 0.5031 | 0.04 | RCFP |
| Bacteria | *Mariprofundus* | 0.2464 | 0.2463 | 0.2309 | 0.2763 | 0.032 | RCFP | Viruses | *Luteoviridae* | 0.2423 | 0.1840 | 0.1829 | 0.3907 | 0.027 | RCFP |
| Bacteria | *Megasphaera* | 0.2244 | 0.2351 | 0.2928 | 0.2478 | 0.022 | CP | Viruses | *Podoviridae* | 0.2796 | 0.1925 | 0.1992 | 0.3286 | 0.018 | RCFP |
| Bacteria | *Methylophaga* | 0.2395 | 0.2464 | 0.2465 | 0.2676 | 0.048 | RCFP | Viruses | *Poxviridae* | 0.1347 | 0.1947 | 0.4710 | 0.1996 | 0.026 | CP |
| Bacteria | *Neptuniibacter* | 0.2459 | 0.2480 | 0.2311 | 0.2750 | 0.005 | RCFP | Viruses | *uc_Tymovirales* | 0.4632 | 0.0996 | 0.1086 | 0.1356 | 0.041 | CCFP |
| Bacteria | *Methylovorus* | 0.2521 | 0.2315 | 0.2386 | 0.2778 | 0.006 | RCFP | Viruses | *Virgaviridae* | 0.2401 | 0.3189 | 0.2234 | 0.2176 | 0.044 | CFP |

Only the microbes of an aquaculture model with the highest indicator values at p < 0.05 were considered indicator microbes for this model. Except for viruses (family level), all indicator microbes were detected at the genus level.

**Table S6. The detailed information of indicator microbes for microbial community across aquaculture models in sediment habitat.**

| Kingdom | Indicator microbes | CCFP | CFP | CP | RCFP | p-value (<0.05) | Culture model | Kingdom | Indicator species | CCFP | CFP | CP | RCFP | p-value (<0.05) | Culture model |
| --- | --- | --- | --- | --- | --- | --- | --- | --- | --- | --- | --- | --- | --- | --- | --- |
| Archaea | *Ferroplasma* | 0.2256 | 0.2445 | 0.2407 | 0.2892 | 0.043 | RCFP | Bacteria | *Kushneria* | 0.2441 | 0.2635 | 0.2765 | 0.2160 | 0.039 | CP |
| Archaea | *Halopiger* | 0.2869 | 0.2393 | 0.2357 | 0.2381 | 0.005 | CCFP | Bacteria | *Leeuwenhoekiella* | 0.2351 | 0.2349 | 0.2500 | 0.2800 | 0.005 | RCFP |
| Archaea | *Halorubrum* | 0.2627 | 0.2547 | 0.2441 | 0.2385 | 0.04 | CCFP | Bacteria | *Limnohabitans* | 0.3425 | 0.2103 | 0.2623 | 0.1849 | 0.037 | CCFP |
| Bacteria | *Actinomadura* | 0.2690 | 0.2482 | 0.2500 | 0.2328 | 0.04 | CCFP | Bacteria | *Luminiphilus* | 0.2365 | 0.2794 | 0.2318 | 0.2522 | 0.01 | CFP |
| Bacteria | *Advenella* | 0.3004 | 0.2206 | 0.2282 | 0.2509 | 0.034 | CCFP | Bacteria | *Mannheimia* | 0.2334 | 0.2475 | 0.2411 | 0.2780 | 0.038 | RCFP |
| Bacteria | *Alcanivorax* | 0.2468 | 0.2636 | 0.2527 | 0.2370 | 0.041 | CFP | Bacteria | *Mobiluncus* | 0.2652 | 0.2507 | 0.2430 | 0.2411 | 0.033 | CCFP |
| Bacteria | *Alicycliphilus* | 0.2964 | 0.2307 | 0.2519 | 0.2209 | 0.006 | CCFP | Bacteria | *Modestobacter* | 0.2895 | 0.2292 | 0.2458 | 0.2355 | 0.009 | CCFP |
| Bacteria | *Alkaliphilus* | 0.2210 | 0.2563 | 0.2338 | 0.2888 | 0.009 | RCFP | Bacteria | *Moraxella* | 0.2300 | 0.2389 | 0.2481 | 0.2830 | 0.011 | RCFP |
| Bacteria | *Alloiococcus* | 0.2347 | 0.2374 | 0.2490 | 0.2789 | 0.034 | RCFP | Bacteria | *Nakamurella* | 0.2810 | 0.2507 | 0.2533 | 0.2150 | 0.019 | CCFP |
| Bacteria | *Anaerolinea* | 0.2457 | 0.2508 | 0.2658 | 0.2377 | 0.03 | CP | Bacteria | *Nitrosomonas* | 0.2368 | 0.2465 | 0.2508 | 0.2659 | 0.017 | RCFP |
| Bacteria | *Anoxybacillus* | 0.2368 | 0.2444 | 0.2270 | 0.2919 | 0.017 | RCFP | Bacteria | *Ornithinimicrobium* | 0.2755 | 0.2515 | 0.2486 | 0.2244 | 0.028 | CCFP |
| Bacteria | *Arthrospira* | 0.2564 | 0.2265 | 0.2367 | 0.2805 | 0.032 | RCFP | Bacteria | *Owenweeksia* | 0.2337 | 0.2367 | 0.2479 | 0.2817 | 0.048 | RCFP |
| Bacteria | *Aureimonas* | 0.2579 | 0.2343 | 0.2822 | 0.2255 | 0.034 | CP | Bacteria | *Pannonibacter* | 0.2570 | 0.2424 | 0.2834 | 0.2172 | 0.027 | CP |
| Bacteria | *Azospirillum* | 0.2776 | 0.2424 | 0.2451 | 0.2348 | 0.004 | CCFP | Bacteria | *Peptoniphilus* | 0.2339 | 0.2344 | 0.2340 | 0.2978 | 0.02 | RCFP |
| Bacteria | *Beutenbergia* | 0.2935 | 0.2362 | 0.2478 | 0.2226 | 0.006 | CCFP | Bacteria | *Phascolarctobacterium* | 0.2401 | 0.2747 | 0.2401 | 0.2450 | 0.005 | CFP |
| Bacteria | *Bibersteinia* | 0.2220 | 0.2450 | 0.2363 | 0.2967 | 0.017 | RCFP | Bacteria | *Polaromonas* | 0.3626 | 0.2349 | 0.1877 | 0.2149 | 0.007 | CCFP |
| Bacteria | *Blastococcus* | 0.2530 | 0.2336 | 0.2783 | 0.2351 | 0.005 | CP | Bacteria | *Pseudanabaena* | 0.1859 | 0.2068 | 0.2522 | 0.3551 | 0.021 | RCFP |
| Bacteria | *Bulleidia* | 0.2387 | 0.2207 | 0.2437 | 0.2968 | 0.023 | RCFP | Bacteria | *Pseudomonas* | 0.2623 | 0.2449 | 0.2516 | 0.2411 | 0.015 | CCFP |
| Bacteria | *Caldanaerobacter* | 0.2419 | 0.2417 | 0.2752 | 0.2412 | 0.028 | CP | Bacteria | *Psychroflexus* | 0.2269 | 0.2374 | 0.2480 | 0.2877 | 0.033 | RCFP |
| Bacteria | *Candidatus_Desulforudis* | 0.2562 | 0.2375 | 0.2704 | 0.2358 | 0.035 | CP | Bacteria | *Rhodopseudomonas* | 0.3038 | 0.2474 | 0.1894 | 0.2594 | 0.037 | CCFP |
| Bacteria | *Candidatus_Hodgkinia* | 0.2966 | 0.2333 | 0.2255 | 0.2446 | 0.017 | CCFP | Bacteria | *Serinicoccus* | 0.2534 | 0.2501 | 0.2799 | 0.2166 | 0.006 | CP |
| Bacteria | *Candidatus_Solibacter* | 0.2693 | 0.2559 | 0.2441 | 0.2307 | 0.037 | CCFP | Bacteria | *Shewanella* | 0.2392 | 0.2402 | 0.2509 | 0.2697 | 0.009 | RCFP |
| Bacteria | *Catenovulum* | 0.2447 | 0.2407 | 0.2297 | 0.2849 | 0.008 | RCFP | Bacteria | *Stenotrophomonas* | 0.3008 | 0.2418 | 0.2453 | 0.2121 | 0.036 | CCFP |
| Bacteria | *Chitinophaga* | 0.2285 | 0.2489 | 0.2481 | 0.2745 | 0.027 | RCFP | Bacteria | *Stigmatella* | 0.2517 | 0.2405 | 0.2775 | 0.2303 | 0.045 | CP |
| Bacteria | *uc_Chloroflexi* | 0.2646 | 0.2430 | 0.2482 | 0.2442 | 0.018 | CCFP | Bacteria | *Streptococcus* | 0.2397 | 0.2424 | 0.2403 | 0.2776 | 0.026 | RCFP |
| Bacteria | *Chloroherpeton* | 0.2686 | 0.2402 | 0.2404 | 0.2508 | 0.027 | CCFP | Bacteria | *Sulfurimonas* | 0.2269 | 0.2440 | 0.2399 | 0.2892 | 0.018 | RCFP |
| Bacteria | *Coleofasciculus* | 0.2325 | 0.2538 | 0.2244 | 0.2893 | 0.022 | RCFP | Bacteria | *Syntrophomonas* | 0.2461 | 0.2762 | 0.2262 | 0.2515 | 0.04 | CFP |
| Bacteria | *Coriobacterium* | 0.2552 | 0.2365 | 0.2731 | 0.2352 | 0.017 | CP | Bacteria | *Thermomonospora* | 0.2632 | 0.2264 | 0.2819 | 0.2285 | 0.048 | CP |
| Bacteria | *Crinalium* | 0.2174 | 0.2571 | 0.2330 | 0.2925 | 0.027 | RCFP | Bacteria | *Truepera* | 0.2698 | 0.2502 | 0.2396 | 0.2405 | 0.019 | CCFP |
| Bacteria | *Desulfotomaculum* | 0.2356 | 0.2501 | 0.2473 | 0.2670 | 0.021 | RCFP | Bacteria | *Veillonella* | 0.2233 | 0.2367 | 0.2451 | 0.2949 | 0.038 | RCFP |
| Bacteria | *Dialister* | 0.2448 | 0.2494 | 0.2434 | 0.2624 | 0.014 | RCFP | Bacteria | *uc_Verrucomicrobia* | 0.2410 | 0.2616 | 0.2482 | 0.2492 | 0.022 | CFP |
| Bacteria | *Enhydrobacter* | 0.2431 | 0.2250 | 0.2353 | 0.2966 | 0.007 | RCFP | Bacteria | *uc_Xanthomonadaceae* | 0.2600 | 0.2858 | 0.2349 | 0.2193 | 0.046 | CFP |
| Bacteria | *Fervidobacterium* | 0.2342 | 0.2431 | 0.2412 | 0.2815 | 0.011 | RCFP | Bacteria | *Xenorhabdus* | 0.2323 | 0.2610 | 0.2271 | 0.2797 | 0.045 | RCFP |
| Bacteria | *Fibrobacter* | 0.2680 | 0.2459 | 0.2351 | 0.2510 | 0.019 | CCFP | Bacteria | *Yersinia* | 0.2448 | 0.2466 | 0.2384 | 0.2702 | 0.005 | RCFP |
| Bacteria | *Fluviicola* | 0.2222 | 0.2260 | 0.2534 | 0.2985 | 0.009 | RCFP | Eukaryota | *Podospora* | 0.2386 | 0.2728 | 0.2474 | 0.2412 | 0.02 | CFP |
| Bacteria | *Glaciecola* | 0.2454 | 0.2457 | 0.2368 | 0.2721 | 0.025 | RCFP | Eukaryota | *Verticillium* | 0.2372 | 0.2769 | 0.2489 | 0.2370 | 0.035 | CFP |
| Bacteria | *Henriciella* | 0.2775 | 0.2342 | 0.2537 | 0.2346 | 0.041 | CCFP | Viruses | *Microviridae* | 0.1416 | 0.2266 | 0.1949 | 0.4370 | 0.018 | RCFP |
| Bacteria | *Ilumatobacter* | 0.2841 | 0.2304 | 0.2635 | 0.2220 | 0.008 | CCFP | Viruses | *Secoviridae* | 0.2554 | 0.2437 | 0.2720 | 0.2288 | 0.023 | CP |
| Bacteria | *Janibacter* | 0.2811 | 0.2583 | 0.2372 | 0.2233 | 0.037 | CCFP | Viruses | *uc_Tymovirales* | 0.0934 | 0.4868 | 0.0311 | 0.1400 | 0.043 | CFP |
| Bacteria | *Kangiella* | 0.2354 | 0.2397 | 0.2411 | 0.2838 | 0.02 | RCFP |  |  |  |  |  |  |  |  |

Only the microbes of an aquaculture model with the highest indicator values at p < 0.05 were considered indicator microbes for this model. Except for viruses (family level), all indicator microbes were detected at the genus level.

**Table S7. The detailed information of indicator microbes for microbial community across aquaculture models in crayfish gut habitat.**

| Kingdom | Indicator microbes | CCFP | CFP | RCFP | p value (<0.05) | Culture model | Kingdom | Indicator species | CCFP | CFP | RCFP | p value (<0.05) | Culture model |
| --- | --- | --- | --- | --- | --- | --- | --- | --- | --- | --- | --- | --- | --- |
| Archaea | *Methanohalophilus* | 0.3188 | 0.3857 | 0.2954 | 0.017 | CFP | Bacteria | *Nocardioides* | 0.1676 | 0.3127 | 0.5197 | 0.031 | RCFP |
| Archaea | *Methanopyrus* | 0.2946 | 0.3057 | 0.3997 | 0.04 | RCFP | Bacteria | *Ornithinimicrobium* | 0.3223 | 0.2531 | 0.4245 | 0.033 | RCFP |
| Bacteria | *Agarivorans* | 0.3308 | 0.2740 | 0.3952 | 0.031 | RCFP | Bacteria | *Parabacteroides* | 0.3077 | 0.3219 | 0.3704 | 0.035 | RCFP |
| Bacteria | *Asticcacaulis* | 0.3061 | 0.3111 | 0.3829 | 0.049 | RCFP | Bacteria | *Prosthecochloris* | 0.3072 | 0.3015 | 0.3913 | 0.015 | RCFP |
| Bacteria | *Beijerinckia* | 0.3160 | 0.2850 | 0.3989 | 0.047 | RCFP | Bacteria | *Pseudonocardia* | 0.2284 | 0.2319 | 0.5397 | 0.005 | RCFP |
| Bacteria | *Candidatus_Koribacter* | 0.3078 | 0.2948 | 0.3974 | 0.039 | RCFP | Bacteria | *Pseudorhodobacter* | 0.3041 | 0.3183 | 0.3776 | 0.04 | RCFP |
| Bacteria | *Cellulomonas* | 0.2933 | 0.2738 | 0.4328 | 0.031 | RCFP | Bacteria | *Pusillimonas* | 0.2639 | 0.2356 | 0.5005 | 0.029 | RCFP |
| Bacteria | *Chloroflexus* | 0.2083 | 0.2257 | 0.5660 | 0.032 | RCFP | Bacteria | *Ralstonia* | 0.4651 | 0.2664 | 0.2685 | 0.024 | CCFP |
| Bacteria | *Chthoniobacter* | 0.2954 | 0.3058 | 0.3988 | 0.03 | RCFP | Bacteria | *Rickettsia* | 0.2851 | 0.2826 | 0.4323 | 0.041 | RCFP |
| Bacteria | *Comamonas* | 0.2185 | 0.3308 | 0.4507 | 0.037 | RCFP | Bacteria | *Rubrobacter* | 0.3064 | 0.3043 | 0.3893 | 0.036 | RCFP |
| Bacteria | *Dichelobacter* | 0.3248 | 0.2935 | 0.3817 | 0.049 | RCFP | Bacteria | *Schlesneria* | 0.2795 | 0.3161 | 0.4044 | 0.02 | RCFP |
| Bacteria | *uc_Firmicutes* | 0.3901 | 0.2849 | 0.3250 | 0.013 | CCFP | Bacteria | *Synechococcus* | 0.3028 | 0.3036 | 0.3936 | 0.037 | RCFP |
| Bacteria | *Gardnerella* | 0.3012 | 0.3156 | 0.3832 | 0.009 | RCFP | Bacteria | *Teredinibacter* | 0.2891 | 0.3259 | 0.3850 | 0.016 | RCFP |
| Bacteria | *Isosphaera* | 0.2979 | 0.3095 | 0.3926 | 0.035 | RCFP | Bacteria | *Thiomonas* | 0.2775 | 0.2401 | 0.4824 | 0.038 | RCFP |
| Bacteria | *Leucobacter* | 0.3037 | 0.2881 | 0.4082 | 0.044 | RCFP | Bacteria | *Tsukamurella* | 0.2925 | 0.2767 | 0.4308 | 0.03 | RCFP |
| Bacteria | *Marichromatium* | 0.2759 | 0.3088 | 0.4153 | 0.015 | RCFP | Bacteria | *Turneriella* | 0.3029 | 0.3044 | 0.3928 | 0.011 | RCFP |
| Bacteria | *Marinobacterium* | 0.3087 | 0.3070 | 0.3843 | 0.036 | RCFP | Bacteria | *Xylella* | 0.2912 | 0.3070 | 0.4018 | 0.049 | RCFP |
| Bacteria | *Maritimibacter* | 0.2673 | 0.3125 | 0.4203 | 0.044 | RCFP | Eukaryote | *Millerozyma* | 0.3362 | 0.3774 | 0.2864 | 0.041 | CFP |
| Bacteria | *Marvinbryantia* | 0.3195 | 0.3246 | 0.3559 | 0.028 | RCFP | Viruses | *Adenoviridae* | 0.5323 | 0.2610 | 0.2067 | 0.019 | CCFP |
| Bacteria | *Meiothermus* | 0.2644 | 0.2749 | 0.4607 | 0.031 | RCFP | Viruses | *Rhabdoviridae* | 0.3349 | 0.3955 | 0.2696 | 0.002 | CFP |
| Bacteria | *Microcystis* | 0.3129 | 0.3063 | 0.3808 | 0.034 | RCFP |  |  |  |  |  |  |  |

Only the microbes of an aquaculture model with the highest indicator values at p < 0.05 were considered indicator microbes for this model. Except for viruses (family level), all indicator microbes were detected at the genus level.

**Table S8. The detailed information for indicator microbes of microbial community across aquaculture models in crab gut habitat.**

| Kingdom | Indicator microbes | CCFP | CP | p-value (<0.05) | Culture model |
| --- | --- | --- | --- | --- | --- |
| Archaea | *Fervidicoccus* | 0.475330236 | 0.5246698 | 0.045 | CP |
| Archaea | *Halovivax* | 0.532251929 | 0.4677481 | 0.077 | CP |
| Bacteria | *Anaerophaga* | 0.466917363 | 0.5330826 | 0.035 | CP |
| Bacteria | *Aquaspirillum* | 0.473254546 | 0.5267455 | 0.007 | CCFP |
| Bacteria | *Carboxydothermus* | 0.447590753 | 0.5524092 | 0.022 | CCFP |
| Bacteria | *Dactylococcopsis* | 0.471967929 | 0.5280321 | 0.024 | CCFP |
| Bacteria | *Dehalobacter* | 0.46652957 | 0.5334704 | 0.029 | CP |
| Bacteria | *Erwinia* | 0.471506823 | 0.5284932 | 0.049 | CP |
| Bacteria | *Fibrisoma* | 0.529554306 | 0.4704457 | 0.02 | CP |
| Bacteria | *Fodinicurvata* | 0.566501239 | 0.4334988 | 0.04 | CCFP |
| Bacteria | *Holdemania* | 0.524499546 | 0.4755005 | 0.036 | CP |
| Bacteria | *Kosmotoga* | 0.4612354 | 0.5387646 | 0.007 | CCFP |
| Bacteria | *Lewinella* | 0.477218534 | 0.5227815 | 0.046 | CP |
| Bacteria | *Methylosarcina* | 0.426470331 | 0.5735297 | 0.032 | CP |
| Bacteria | *Methylovulum* | 0.554264875 | 0.4457351 | 0.028 | CCFP |
| Bacteria | *uc_Rhodobacterales* | 0.477835794 | 0.5221642 | 0.034 | CCFP |
| Bacteria | *Rickettsiella* | 0.449606295 | 0.5503937 | 0.046 | CP |
| Bacteria | *uc_Vibrionales* | 0.466337648 | 0.5336624 | 0.033 | CP |
| Eukaryota | *Coccidioides* | 0.436805143 | 0.5631949 | 0.003 | CP |
| Eukaryota | *Magnaporthe* | 0.469981629 | 0.5300184 | 0.036 | CP |
| Eukaryota | *Phaeodactylum* | 0.474759326 | 0.5252407 | 0.038 | CP |
| Eukaryota | *Uncinocarpus* | 0.478381945 | 0.5216181 | 0.027 | CP |
| Viruses | *Microviridae* | 0.694140181 | 0.3058598 | 0.047 | CP |
| Viruses | *Permutotetraviridae* | 0.680503881 | 0.3194961 | 0.03 | CP |

Only the microbes of an aquaculture model with the highest indicator values at p < 0.05 were considered indicator microbes for this model. Except for viruses (family level), all indicator microbes were detected at the genus level.

**Table S9. Network properties for RCFP and non-RCFP in water, sediment, and crayfish gut habitats, respectively.**

| Network properties | water | |  | sediment | |  | crayfish gut | |
| --- | --- | --- | --- | --- | --- | --- | --- | --- |
|  | RCFP | non-RCFP |  | RCFP | non-RCFP |  | RCFP | non-RCFP |
| Node number | 40 | 24 |  | 42 | 26 |  | 46 | 49 |
| Edge number | 177 | 33 |  | 231 | 26 |  | 501 | 153 |
| Node average degree | 8.85 | 2.75 |  | 11 | 2 |  | 21.78 | 6.25 |
| Network density | 0.11 | 0.06 |  | 0.13 | 0.04 |  | 0.24 | 0.07 |
